# Serum surfactant protein D as a significant biomarker for predicting occurrence, progression, acute exacerbation, and mortality in interstitial lung disease: a systematic review and meta-analysis

**DOI:** 10.3389/fimmu.2025.1450798

**Published:** 2025-02-14

**Authors:** Xing He, Jiaqi Ji, Dan Zheng, Zeli Luo, Linjie Luo, Lu Guo

**Affiliations:** ^1^ Department of Pulmonary and Critical Care Medicine, West China Hospital, Sichuan University, Chengdu, China; ^2^ State Key Laboratory of Respiratory Health and Multimorbidity, West China Hospital, Sichuan University, Chengdu, Sichuan, China; ^3^ Department of Pulmonary and Critical Care Medicine, Sichuan Provincial People’s Hospital, School of Medicine, University of Electronic Science and Technology of China, Chengdu, China; ^4^ Department of Critical Care Medicine, Wenjiang District People’s Hospital of Chengdu, Chengdu, China

**Keywords:** surfactant protein D, interstitial lung disease, occurrence, progression, acute exacerbation, mortality

## Abstract

**Objective:**

Serum surfactant protein D (SP-D) is a potential biomarker for the non-invasive prediction of interstitial lung disease (ILD) status. However, previous studies lacked comprehensively qualitative and quantitative pooled analysis methods to summarize the relationship between SP-D and ILD.

**Methods:**

We conducted a comprehensive literature search from PubMed, Embase, Web of Science, Scopus, Ovid, and Cochrane Library, up to 16 December 2023. The Newcastle–Ottawa Quality Assessment Scale was employed to evaluate the quality of each included study. Pooled analyses were primarily performed for weighted mean difference (WMD), odds ratio (OR), and hazard ratio (HR). Sensitivity analysis was conducted by sequentially eliminating one study at a time and reanalyzing the remaining studies. In addition, the trim-and-fill method was applied for correcting publication bias.

**Results:**

More than 3,561 patients with ILD from 41 articles were included for pooled analysis. The pooled results showed that serum SP-D levels were higher in the ILD group than the control group (WMD = 120.24 ng/mL, 95% CI: 72.45–168.03, p<0.001). Additionally, SP-D levels among patients with ILD were significantly elevated in the acute exacerbation (AE) group compared with the non-AE group (WMD = 9.88 ng/mL, 95% CI: 2.64–17.12, p=0.008), and in the death group compared with the survival group (WMD = 32.98 ng/mL, 95% CI: 2.11–63.84, p=0.036). However, no significant difference was observed between the progression group and the stable group (WMD = 13.54 ng/mL, 95% CI: −23.68–50.76, p=0.227). In addition, pooled results demonstrated that serum SP-D was a reliable predictive factor for various outcomes associated with ILD: occurrence (OR=4.66, 95%CI = 2.46, 8.86, p<0.001), progression (OR=1.003, 95%CI= 1.001, 1.006, p=0.033), and mortality (HR=1.002, 95%CI= 1.001, 1.003, p=0.023) of ILD. In contrast, there was no significant difference for predicting AE (HR = 1.004, 95% CI = 0.997, 1.011, p=0.240).

**Conclusion:**

Serum SP-D is a significant biomarker associated with ILD occurrence, progression, acute exacerbation, and mortality. It remains essential to clarify the predictive value of serum SP-D levels concerning the disease status in patients with different ILD subtypes. Moreover, it may be beneficial to conduct a combined analysis of SP-D with other potential biomarkers to further enhance its diagnostic capability regarding the disease status in patients with ILD.

**Systematic Review Registration:**

https://inplasy.com/inplasy-2024-5-0050/, identifier INPLASY 202450050.

## Introduction

Interstitial lung disease (ILD) is a broad term that encompasses a heterogeneous group of diseases, including idiopathic interstitial pneumonitis, connective tissue disease-associated interstitial lung disease (CTD-ILD), sarcoidosis, and some pulmonary rare diseases. The pathologic manifestations of ILD are characterized by inflammation and fibrosis, leading to patients experiencing varying degrees of cough and dyspnea. Some studies have shown that the incidence of ILD ranges from 1 to 31.5 cases per 100,000 individuals annually, and its prevalence varies between 6.3 and 71 cases per 100,000 people (1). Approximately 34% patients with ILD exhibit progressive fibrosing features (2). During a 5-year follow-up period, acute exacerbation (AE) occurs in approximately 20% of ILD cases; additionally, a mortality rate of 23.2% has been observed over an 11-year follow-up period (3, 4). However, there is a lack of effective prediction methods for assessing risk events associated with ILD. It is important to identify potential biomarkers that can facilitate the recognition of state changes in patients with ILD.

As a glycoprotein, surfactant protein D (SP-D) is primarily synthesized and secreted by alveolar type II epithelial cells, exhibiting anti-infectious and immunomodulatory functions (5). During persistent lung injury, serum SP-D levels are significantly elevated (6). SP-D plays a crucial role in modulating the number of macrophages and fibroblasts within the lung, influencing the expression of profibrotic cytokines, and contributing to pulmonary fibrosis remodeling (7). It has been established that SP-D is an important member in the process of lung inflammation and fibrosis (8, 9), serving as a non-invasive biomarker for evaluating the pulmonary pathological status in ILD (10).

Although serum SP-D has been widely used in the evaluation for diagnosis, treatment, and prognosis across different types of ILD, there still remains controversy about its predictive ability in patients with ILD. Therefore, we conducted this meta-analysis to provide reliable evidence for elucidating the clinical significance of serum SP-D in patients with ILD.

## Materials and methods

The study was in accordance with the Preferred Reporting Items for Systematic Reviews and Meta-Analyses (PRISMA) guidelines (11) and registered with INPLASY (http://INPLASY.com) under registration number INPLASY 202450050.

### Search strategy

A systematic literature search was performed across multiple databases, including PubMed, Embase, Web of Science, Scopus, Ovid, and Cochrane Library, up to December 16, 2023. The primary search terms were “surfactant protein D,” “SP-D,” “interstitial lung disease,” and “ILD” (**Supplementary Table 1**).

### Eligibility criteria

The inclusion criteria were as follows: (1) cohort studies (prospective or retrospective) and cross-sectional studies; (2) ILD was diagnosed principally according to established clinical guidelines (12–15), clinical features and high-resolution computed tomography (HRCT); a pathological confirmation was required when necessary. AE was defined as a deterioration of respiratory symptoms accompanied by new bilateral ground-glass opacification or consolidation, which could not be attributed to infection, heart failure, or other identifiable causes (16–18); progression was defined as a decline in forced vital capacity (FVC) ≥5% predicted and/or diffusing lung capacity for carbon monoxide (DLCO) ≥10% predicted within 1 year of follow-up; (3) availability of quantitative continuous variable data or the ability to convert the data by algorithms; (4) hazard ratio (HR) was calculated by the Cox proportional hazard model, and odds ratio (OR) was analyzed by the logistic regression model; (5) serum SP-D was included as a study parameter; (6) English literature.

The exclusion criteria were as follows: (1) review/meta-analysis, case report, letter, comment, conference abstract, and animal/cell study; (2) ILD patients with lung cancer; (3) laboratory test results for SP-D were not from serum samples; (4) lack of extracted effect sizes for pooled analysis.

### Quality assessment (risk of bias) and data extraction

Two investigators (XH, JJ) independently reviewed all literature that met the inclusion criteria, whereas ZL and LL evaluated the quality of studies through Newcastle–Ottawa Quality Assessment Scale (NOS) (19). The NOS is a widely utilized tool for assessing the quality of case–control and cohort studies. It evaluates study quality through three major modules comprising a total of eight items. These items specifically address the study population selection, comparability, and assessment of exposure/outcome. The total score of NOS ranges from 0 to 9 “stars,” with higher scores indicating higher quality of included studies: 7–9 “stars” signifying high quality, 4–6 “stars” indicating moderate quality, 0–3 “stars” reflecting low quality. The extracted data included the following variables: the first author, year of publication, country, study type, ILD type, comparative group, age, gender, smoking status, KL-6 level, FVC%, DLCO%, detection method of SP-D, sample size or effect size, mean ± standard deviation (SD), odds ratio (OR), hazard ratio (HR), and 95% CI for OR and HR respectively. Study events encompassed occurrence, AE, progression, and mortality associated with ILD. If any disputation in the process, it could be discussed with the arbitrator (LG).

### Data synthesis

The weighted mean difference (WMD) was calculated from extraction data (mean ± SD) for pooled analysis, whereas OR (95% CI) and HR (95% CI) of extraction data were pooled for analysis following log transformation. All studies that performed pooled analysis were initially tested for heterogeneity using Cochran’s Q statistic and inconsistency value (I^2^). If a p-value of <0.05 or I^2^ ≥50% indicated remarkable heterogeneity, a random-effect model and the DerSimonian–Laird (DL) method were ultimately employed to synthesize the data. Meta-regression analysis was conducted to identify potential sources of heterogeneity, and subgroup analysis was applied for further elucidation. For groups without significant heterogeneity, a fixed-effect model and inverse-variance (IV) method were utilized. Subgroup analysis regarding ILD occurrence was performed according to the type of control group. Excluding one category of literature at a time method was implemented for sensitivity analysis; if the exclusion of any category did not significantly affect the results, it suggested that our findings were stable and reliable. Publication bias was judged by Egger’s test; if p<0.05, the trim-and-fill method would be employed for bias correction. Stata software (package meta, version 16.0) was used for statistical analysis, with p<0.05 indicating statistically significant.

## Results

### Study selection and characteristics

**Figure 1:**
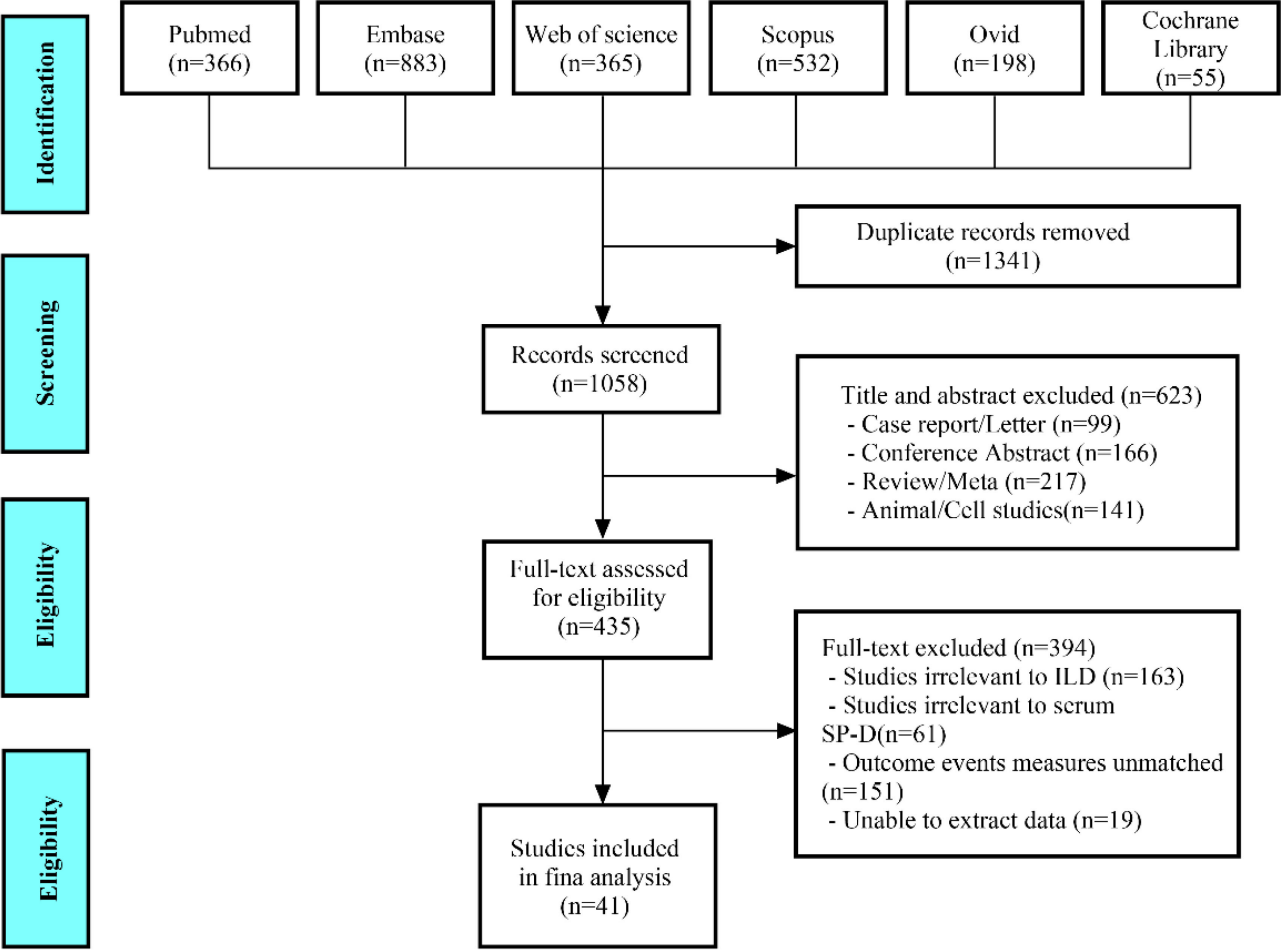
Diagram of the preferred reporting items for systematic review and meta-analysis (PRISMA).

A comprehensive search identified a total of 2,399 studies from the following databases: PubMed (n=366), Embase (n=883), Web of Science (n=365), Scopus (n=532), Ovid (n=198), and Cochrane Library (n=55). After removing 1,341 duplicate records, 1,058 studies remained for title and abstract screening. Of these, 99 studies were excluded as case reports or letters, 166 as conference abstracts, 21 as reviews or meta-analyses, and 141 as studies involving animals or cells. The full texts of the remaining 435 articles were reviewed; among them, 163 studies were unrelated to ILD, 61 studies did not focus on SP-D, 151 studies were irrelevant to the observation events of our research, and 19 studies were unable to extract original data. Ultimately, after rigorous full-text screening in accordance with the PRISMA guidelines (**Figure 1**), a total of 41 studies encompassing more than 3,561 patients with ILD were finally included for pooled analysis. The ILD population in this research originated from various countries, including Japan (n= 29), Greece (n=2), South Korea (n=2), Poland (n=1), Ireland (n=1), China (n=1), America (n=1), Netherlands (n=1), Australia (n=1), India (n=1), and France/Japan/Switzerland (n=1). A total of 12 studies revealed the relationship between serum SP-D levels and the occurrence of ILD (20–31), five studies investigated the significance of serum SP-D in ILD progression (32–36), nine studies explored the clinical value of SP-D in AE-ILD (37–45), and 17 studies reported the serum SP-D levels in patients with ILD between the survival and death groups (32, 44, 46–60). The NOS score showed that 39 studies were considered high quality; two studies were classified as being of moderate quality, primarily due to uncertainties regarding intergroup comparability and unspecified non-response rates (**Supplementary Table 2**). More detailed information about each included study is provided in **Supplementary Tables 3**-**10**.

### Pooled analysis for occurrence of ILD

**Figure 2:**
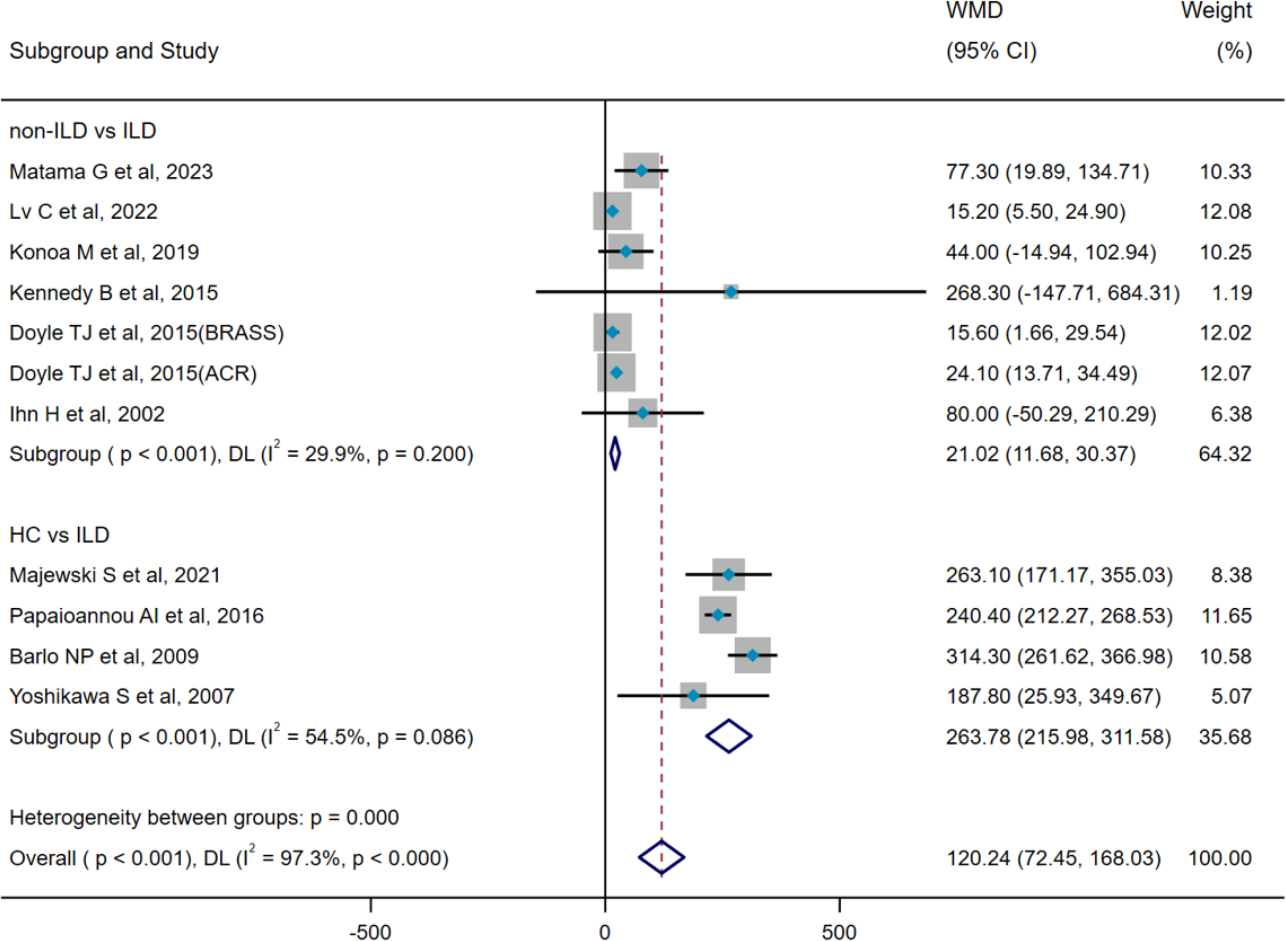
Pooled analysis of WMD (95% CI) in serum SP-D levels between ILD and HC and non-ILD (X-axis).

**Figure 3:**
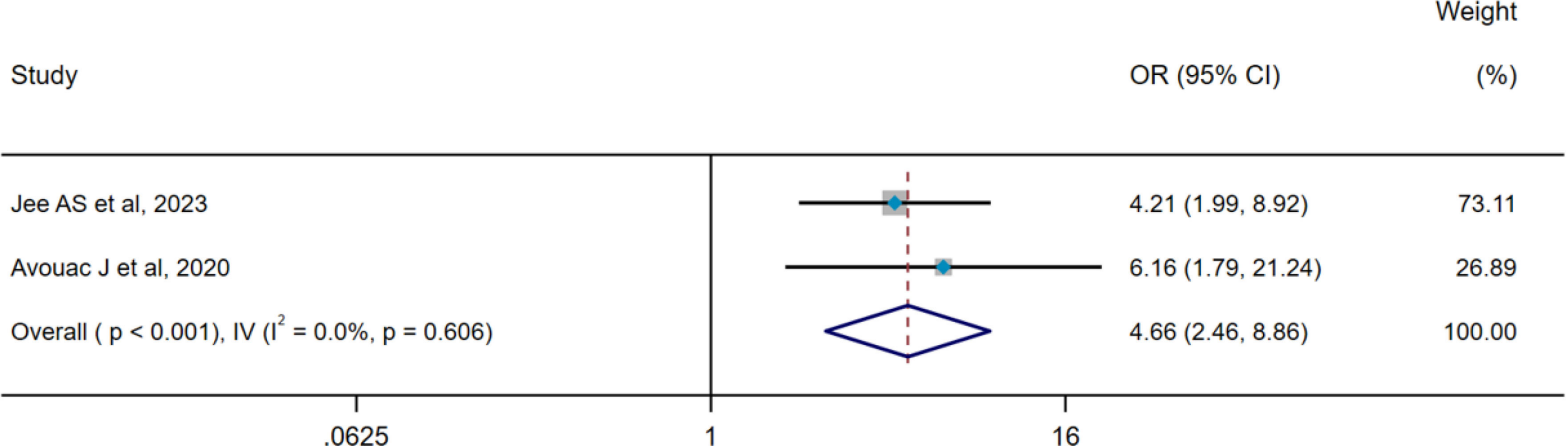
Pooled analysis of the OR for serum SP-D predicting the occurrence of ILD (X-axis). The OR was calculated using multivariate logistic regression analysis, with adjustments made for age and sex. ILD, interstitial lung disease; SP-D, surfactant protein D; OR, odds ratio; IV, inverse-variance method; CI, confidence interval.

There was significant heterogeneity among the 10 included studies (pooled WMD, I^2^ = 97.3%, p<0.001), so the DL method and a random-effect model were used for pooled analysis. Pooled results indicated that serum SP-D levels were significantly higher in the ILD group compared with the control groups (WMD = 120.24 ng/mL, 95% CI: 72.45-168.03, p<0.001) (**Figure 2**). To identify the source of heterogeneity, we conducted meta-regression analyses, suggesting that variations in control groups may contribute to this heterogeneity (p < 0.001)(**Supplementary Figure 2**). Subsequently, we further performed subgroup analyses based on the type of control groups. Subgroup analyses showed that serum SP-D levels in the ILD group were significantly higher than in the non-ILD group (WMD= 21.02 ng/mL, 95% CI: 11.68-30.37, p<0.001), whereas serum SP-D levels in the ILD group were remarkably higher compared with the healthy control (HC) group (WMD= 263.78 ng/mL, 95% CI: 215.98-311.58, p<0.001) (**Figure 2**). No heterogeneity was observed in two studies assessing the pooled OR of ILD occurrence (pooled OR, I^2^ = 0%, p=0.606), so the IV method and a fixed-effect model were utilized for the analysis; the results demonstrated that serum SP-D was a potential risk factor for ILD occurrence (OR=4.66, 95%CI= 2.46, 8.86, p<0.001) (**Figure 3**).

In studies assessing the occurrence of SP-D and ILD, we conducted a subgroup analysis based on the type of control groups included in each study. This was categorized as follows: (1) non-ILD vs. ILD: comparing disease control groups without ILD to those with ILD (e.g., CTD versus CTD-ILD); (2) HC vs. ILD: contrasting healthy populations against those with ILD (e.g., HC versus hypersensitivity pneumonia).

ILD, interstitial lung disease; CTD, connective tissue disease; HC, healthy control; SP-D, surfactant protein D; WMD, weighted mean difference; CI, confidence interval; DL, DerSimonian–Laird method.

### Pooled analysis for progression of ILD

**Figure 4:**
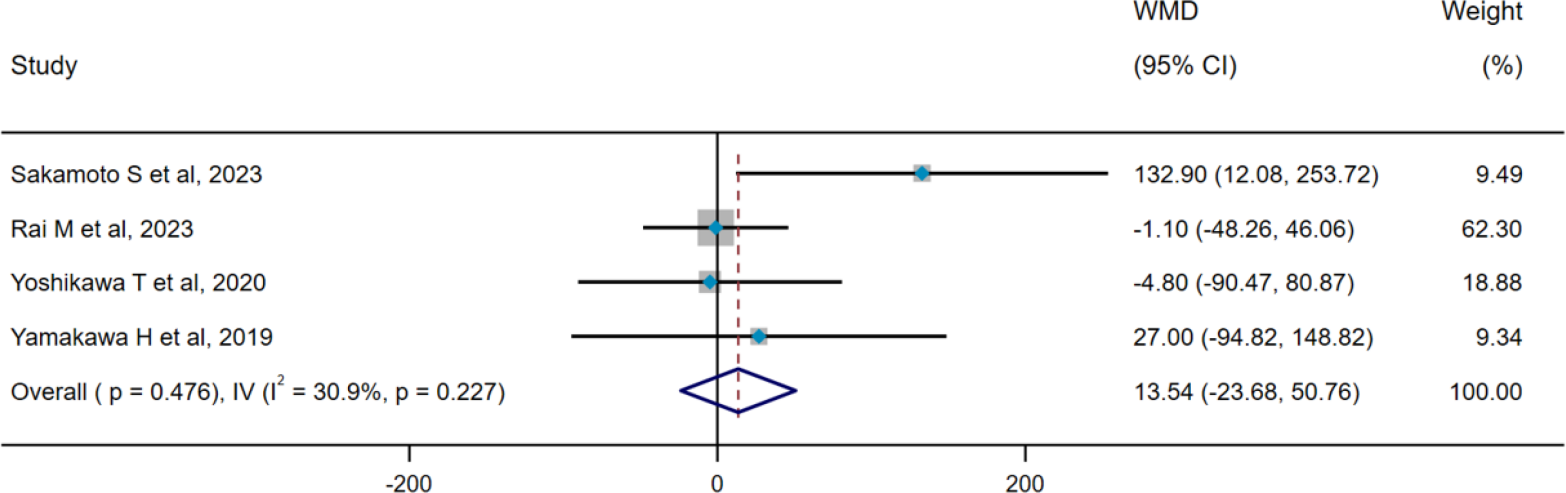
Pooled analysis of WMD (95% CI) in serum SP-D levels between progression group and stable group (X-axis). SP-D, surfactant protein D; WMD, weighted mean difference; IV, inverse-variance; CI, confidence interval.

**Figure 5:**
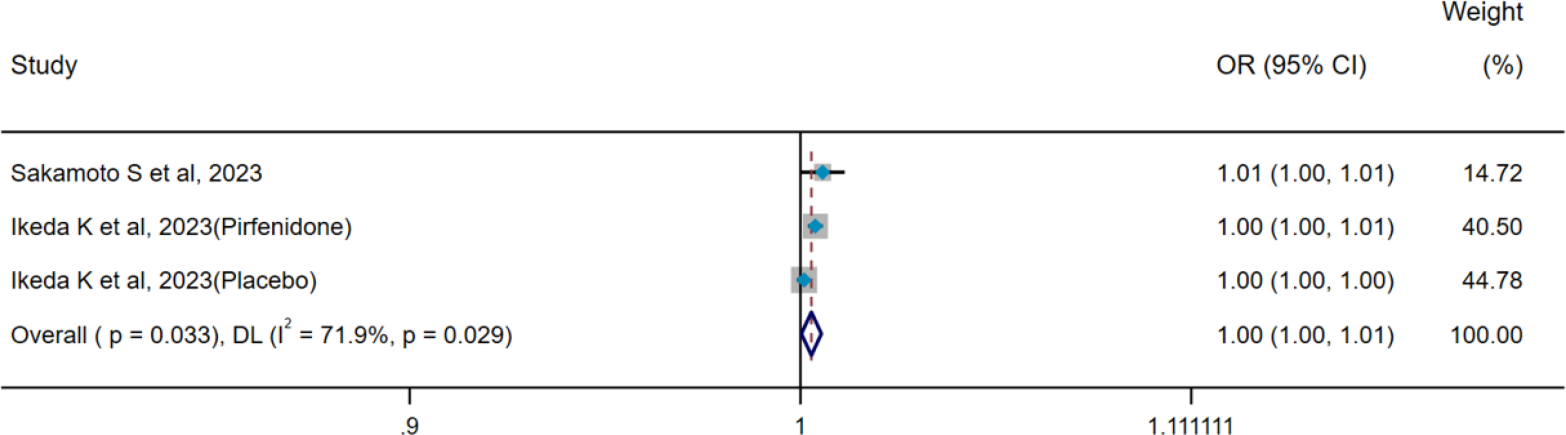
Pooled analysis of the OR for serum SP-D predicting the progression of ILD (X-axis). The OR was calculated using univariate logistic regression analysis. ILD, interstitial lung disease; SP-D, surfactant protein D; OR, odds ratio; DL, DerSimonian–Laird; CI, confidence interval.

There was no heterogeneity among the four included studies (pooled WMD, I^2^ = 30.9%, p=0.227), so the IV method and a fixed-effect model were used for pooled analysis. The pooled result indicated that there was no difference in serum SP-D levels between the progression group and stable group (WMD = 13.54 ng/mL, 95% CI: −23.68-50.76, p=0.476) (**Figure 4**). Two studies included in pooled analysis (pooled OR, I^2^ = 71.9%, p=0.029) exhibited significant heterogeneity, prompting the use of the DL method and a random-effect model for subsequent analyses. Results suggested that serum SP-D may serve as a potential risk factor for ILD progression (OR=1.003, 95%CI= 1.001, 1.006, p=0.033) (**Figure 5**).

### Pooled analysis for acute exacerbation of ILD

**Figure 6:**
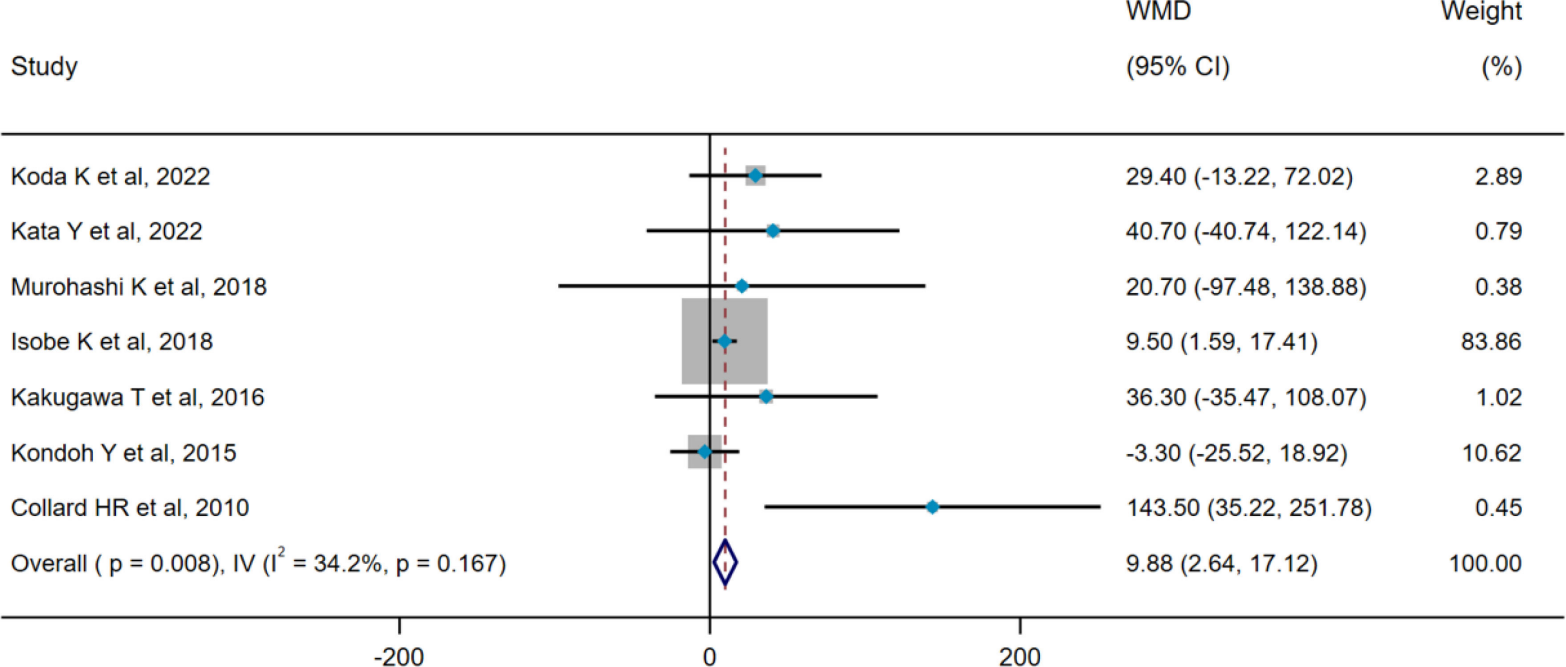
Pooled analysis of WMD (95% CI) in serum SP-D levels between the non-AE group and the AE group (X-axis). SP-D, surfactant protein D; AE, acute exacerbation; WMD, weighted mean difference; IV, inverse-variance; CI, confidence interval.

**Figure 7:**
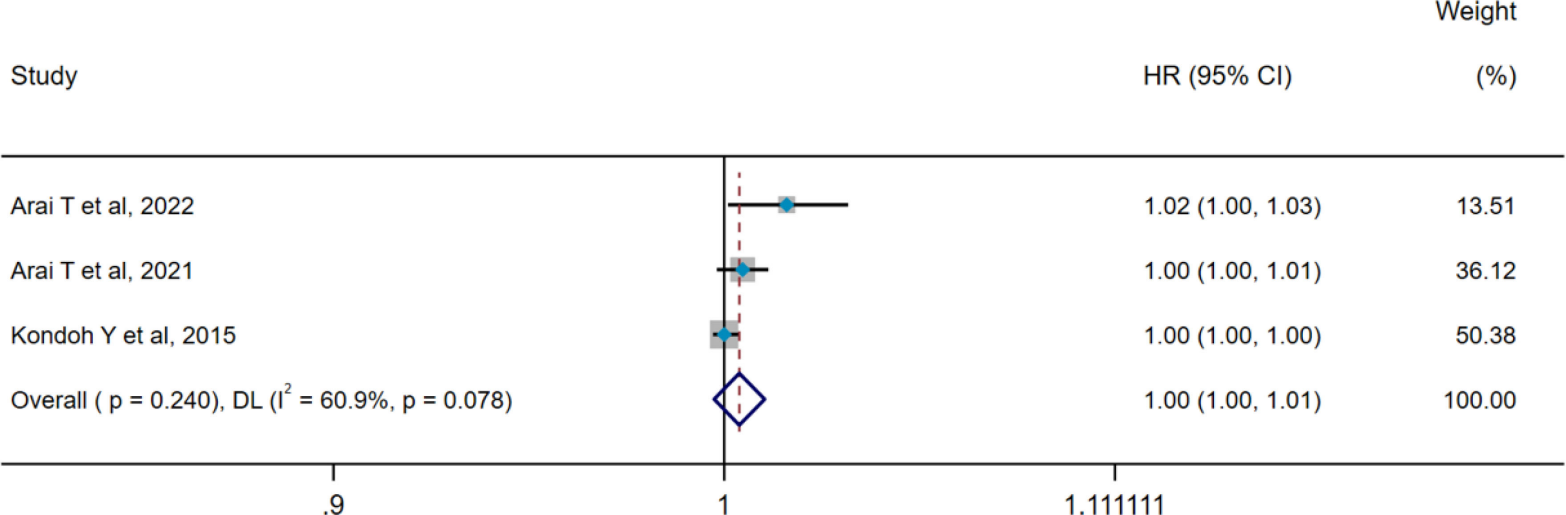
Pooled analysis of the HR for serum SP-D predicting AE in patients with ILD (X-axis). The HR was calculated using univariate Cox proportional hazard regression. ILD, interstitial lung disease; SP-D, surfactant protein D; AE, acute exacerbation; HR, hazard ratio; DL, DerSimonian–Laird; CI, confidence interval.

Heterogeneity results revealed no heterogeneity among seven studies (pooled WMD, I^2^ = 34.2%, p=0.167), so the IV method and a fixed-effect model were applied for pooled analysis. Pooled results showed that serum SP-D levels were significantly higher in the AE group compared with the non-AE group (WMD = 9.88 ng/mL, 95%CI: 2.64-17.12, p=0.008) (**Figure 6**). Three studies concerning AE-ILD displayed heterogeneity (pooled HR, I^2^ = 60.9%, p=0.078), so the DL method and a random-effect model were selected for pooled analysis. There was no significant difference in serum SP-D for predicting AE (HR=1.004, 95%CI= 0.997, 1.011, p=0.240) (**Figure 7**).

### Pooled analysis for mortality of ILD

**Figure 8:**
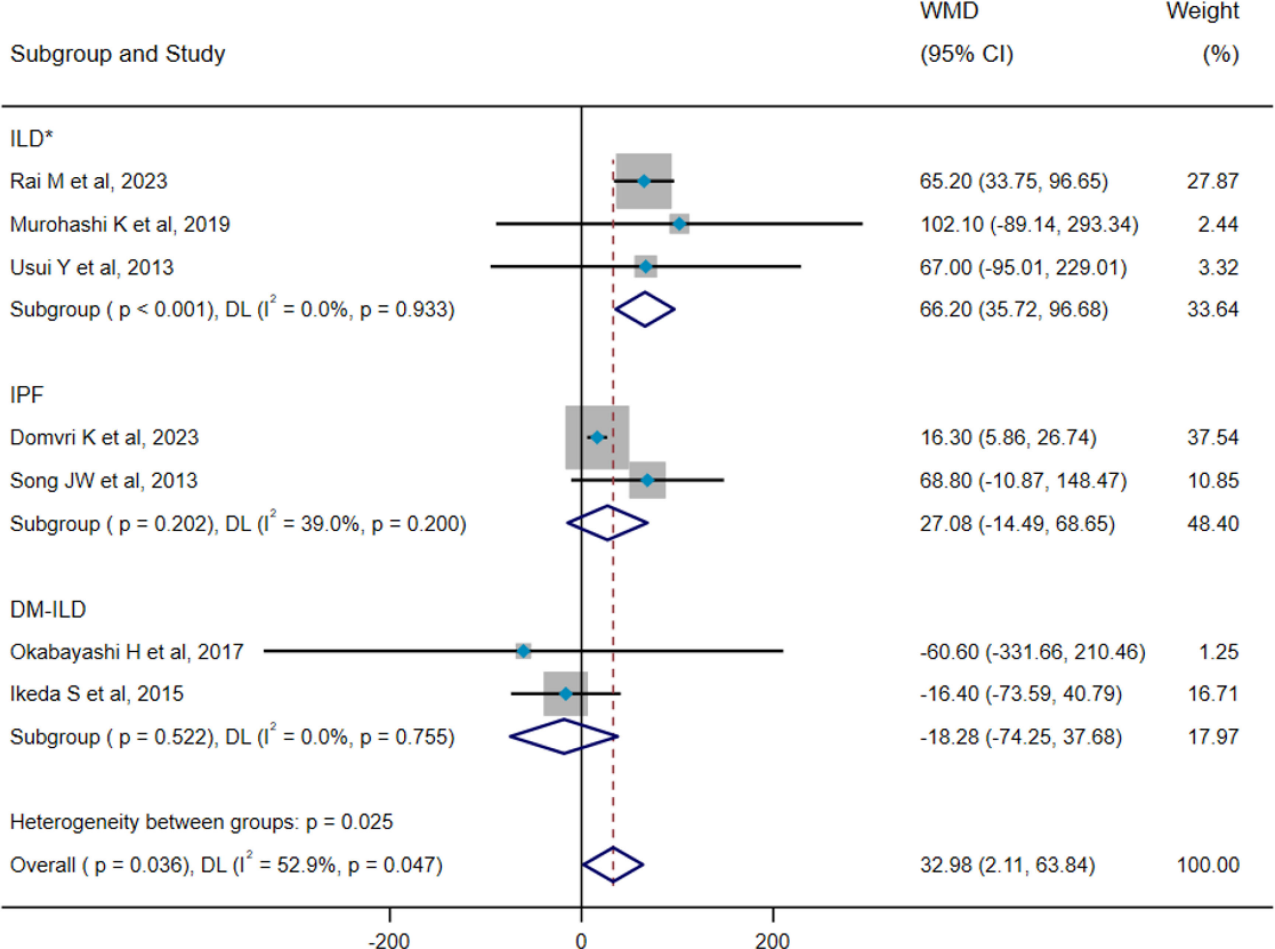
Pooled analysis of WMD (95% CI) in serum SP-D levels between survival group and death group (X-axis). *The included studies did not distinguish the precise subtype of ILD. ILD, interstitial lung disease; IPF, idiopathic pulmonary fibrosis; DM-ILD, dermatomyositis-associated interstitial lung disease; SP-D, surfactant protein D; WMD, weighted mean difference; DL, DerSimonian–Laird; CI, confidence interval.

**Figure 9:**
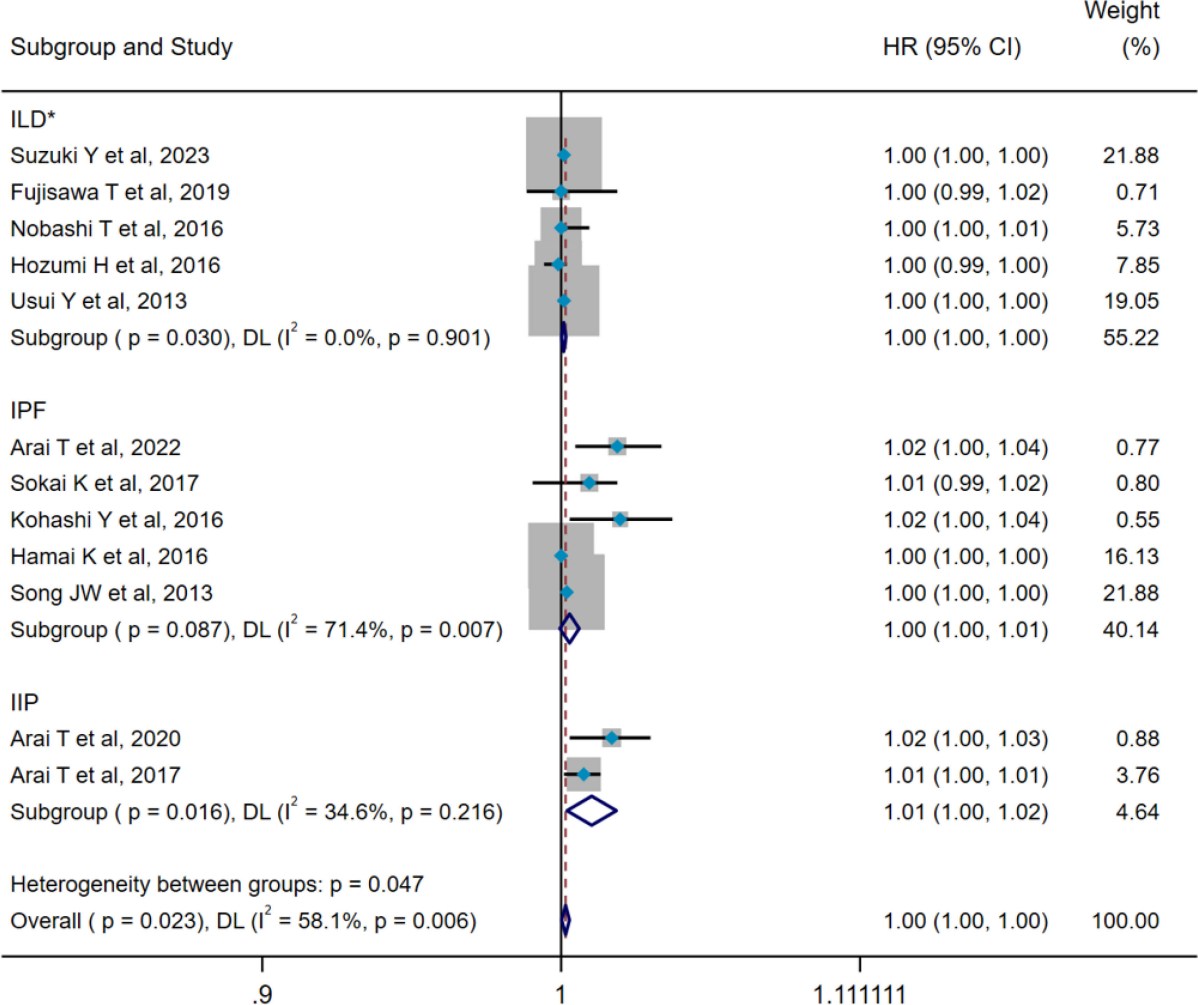
Pooled analysis of the HR for serum SP-D predicting the mortality in patients with ILD (X-axis). The HR was calculated using univariate Cox proportional hazard regression. The included studies did not distinguish the precise subtype of ILD. *The included studies did not distinguish the precise subtype of ILD. ILD, interstitial lung disease; IPF, idiopathic pulmonary fibrosis; IIP, idiopathic interstitial pneumonitis; SP-D, surfactant protein D; HR, hazard ratio; DL, DerSimonian–Laird; CI, confidence interval.

The results of heterogeneity tests indicated that both 7 studies (pooled WMD, I^2^ = 52.9%, p=0.047) and 12 studies (pooled HR, I^2^ = 58.1%, p=0.006) showed remarkably heterogeneity; thus, the DL method and a random-effect model were employed for pooled analysis. Pooled results demonstrated that death group showed higher serum SP-D levels compared with the survival group (WMD = 32.98 ng/mL, 95% CI: 2.11-63.84, p=0.036) (**Figure 8**). Furthermore, serum SP-D could serve as a potential predictor for mortality (HR=1.002, 95%CI= 1.001, 1.003, p=0.023) (**Figure 9**). To explore the source of heterogeneity, we conducted a meta-regression analysis on the pooled WMD of all included studies. The findings suggested that different subtypes of ILD may be potential contributors to the heterogeneity (p=0.025) (**Supplementary Figure 9**). Subgroup analyses reported that serum SP-D levels in the death group were significantly higher than those in the survival group (WMD = 66.20 ng/mL, 95% CI: 35.72-96.68, p<0.001). However, no statistically significant differences in serum SP-D levels were observed between the death and survival groups for idiopathic pulmonary fibrosis (IPF) (WMD = 27.08 ng/mL, 95% CI: −14.49-68.65, p=0.202) and dermatomyositis-associated ILD (WMD = −18.28 ng/mL, 95% CI: −74.25-37.68, p=0.522) (**Figure 8**).

Additionally, subgroup analyses indicated that serum SP-D could act as a predictive factor for mortality among patients with ILD (HR=1.001, 95%CI= 1.000, 1.002, p=0.030) and idiopathic interstitial pneumonia (HR=1.011, 95%CI= 1.002, 1.020, p=0.016), but not for poor prognosis in IPF (HR=1.003, 95%CI= 1.000, 1.006, p=0.087) (**Figure 9**). Moreover, significant heterogeneity was noted within the IPF group. A further meta-regression analysis revealed that factors such as age, sample size, male gender, FVC%, and DLCO% did not emerge as statistically significant sources of heterogeneity within the group (**Supplementary Table 11**).

### Sensitivity analysis and publication bias

**Figure 10:**
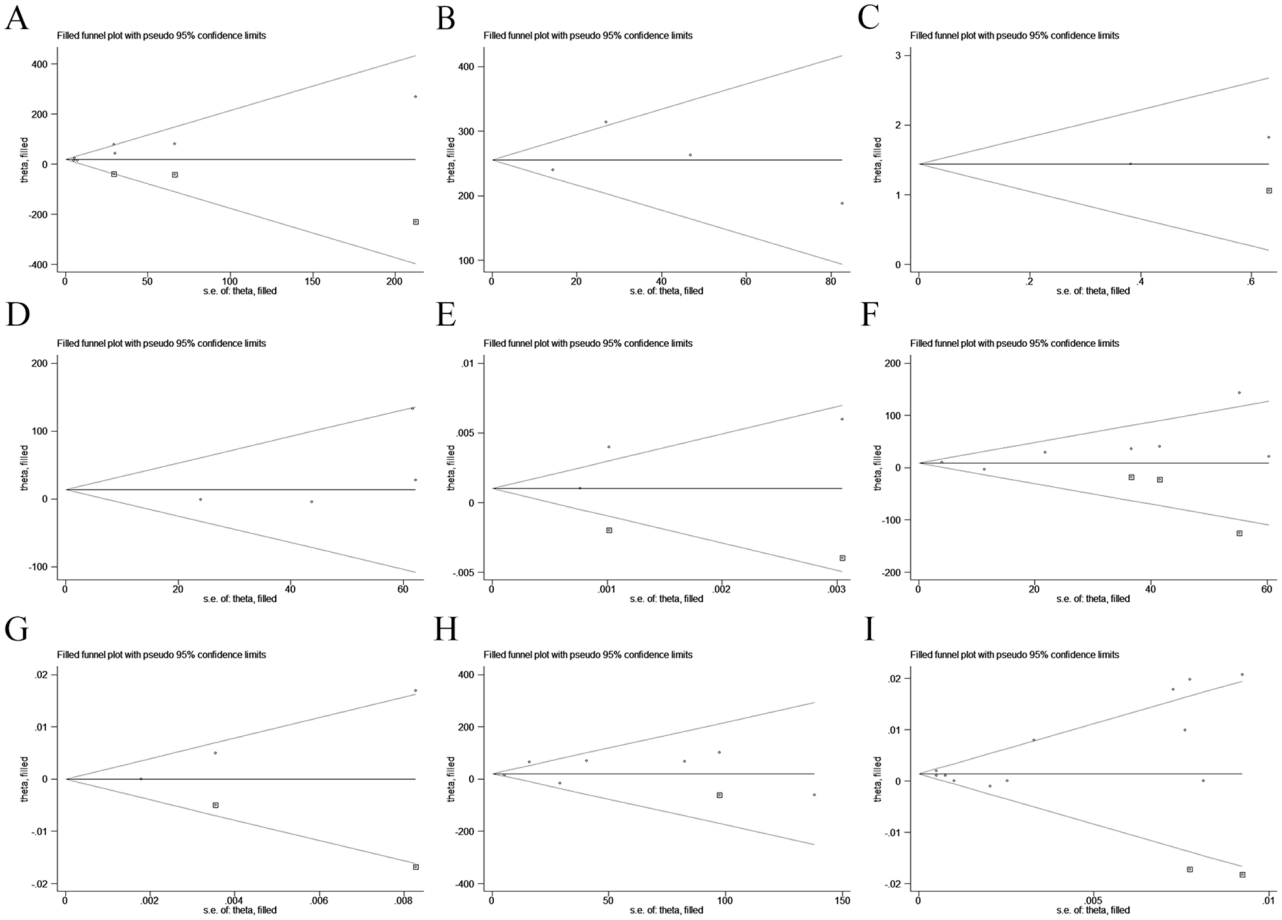
Funnel plots for the symmetry and publication bias of comparative meta-analyses regarding the relationship between serum SP-D and the occurrence, progression, AE, and mortality of ILD. **(A)** non-ILD vs. ILD (pooled WMD); **(B)** HC vs. ILD (pooled WMD); **(C)** occurrence (pooled OR); **(D)** stable vs. progression (pooled WMD); **(E)** progression (pooled OR); **(F)** non-AE vs. AE (pooled WMD); **(G)** AE (pooled HR); **(H)** survival vs. death (pooled WMD); **(I)** AE (pooled HR). ILD, interstitial lung disease; SP-D, surfactant protein D; AE, acute exacerbation.

The results of sensitivity analyses demonstrated that our results were stable (**Supplementary Figures 1**-**8**). Egger’s test showed potential publication bias between non-ILD and ILD groups (p < 0.05) (**Table 1**). Considering the limitations of Egger’s test when the number of studies being pooled was small, we subsequently performed a trim-and-fill method for adjustment, which did not alter the statistical results. When some studies were respectively added to the pooled analyses of included groups, no publication bias was observed in these categories (**Figure 10**) (**Table 1**).

**Table 1:** Egger’s test, Metatrim-filled study, and publication bias test for included studies on serum SP-D predicting occurrence, progression, AE, and mortality in patients with ILD.

Group	n study	Egger’s test		Metatrim-filled study	Publication bias
		t	*P*		
Non-ILD vs. ILD (WMD)	6	3.04	0.029	3	No
HC vs. ILD (WMD)	4	0.23	0.837	0	No
Non-ILD + HC vs. ILD (WMD)	10	2.11	0.064	NA^#^	No
Occurrence (OR, multivariable)	2	NA	NA	1	No
Stable vs. progression (WMD)	4	1.32	0.317	0	No
Progression (OR, univariable)	2	NA	NA	2	No
Non-AE vs. AE (WMD)	7	1.73	0.144	3	No
AE (HR, univariable)	3	NA	NA	2	No
Survival vs. death (WMD)	7	0.83	0.445	1	No
Mortality (HR, univariable)	12	2.01	0.072	2	No

^#^Due to the heterogeneity, we do not show the trim-and-fill method analysis; NA, not acquire; ILD, interstitial lung disease; SP-D, surfactant protein D; AE, acute exacerbation; WMD, weighted mean difference; HC, healthy control; OR, odds ratio; HR, hazard ratio.

## Discussion

Exploring noninvasive methods to assess the condition and prognosis of patients with ILD has emerged as a prominent topic in ILD-related research. ILD encompasses a variety of lung diseases with different etiologies, making the identification of predictive markers for ILD status a challenging endeavor. Serum SP-D has been confirmed to be a potential biomarker for ILD, as reported in numerous studies (36, 61, 62). However, there is a lack of pooled analysis to provide more reliable evidence. Our study comprehensively summarized the clinical significance of serum SP-D in assessing ILD status through both quantitative and qualitative pooled analyses.

SP-D is an active protein involved in modulating pulmonary inflammatory responses (63), and its serum levels are significantly increased in patients with acute and chronic lung injury (64). Notably, serum SP-D levels increase progressively with the exacerbation of ILD (61). Previous studies had suggested that SP-D could serve as a valuable marker for diagnosing and prognosticating IPF (61), as well as monitoring the activity and severity of myositis-associated ILD (65). Nevertheless, some studies have yielded results that contradict this perspective. For example, Kondoh et al. found that serum SP-D levels were higher in stable IPF than AE-IPF (42). Additionally, another meta-analysis conducted by Hannah et al. pointed out that serum SP-D could not reliably predict mortality risk in idiopathic inflammatory myopathy-associated ILD (IIM-ILD) (66). Therefore, our study provided credible evidence for clarifying the relationship between SP-D and various statuses within ILD.

As demonstrated by our pooled analysis, serum SP-D levels were higher in patients with ILD compared with those with non-ILD or healthy populations. Furthermore, serum SP-D could serve as an early predicting marker for the occurrence of ILD. Doyle1 et al. confirmed a significant correlation between serum SP-D levels and the diagnosis of rheumatoid arthritis-associated ILD (21), whereas Okamoto et al. reported that SP-D played an important role in diagnosing hypersensitivity pneumonitis (67); their findings align with ours. It is noteworthy that in our study, the mean serum SP-D levels in IPF and systemic sclerosis-associated ILD (SSc-ILD) were higher than those observed in SSc or healthy controls (20, 23, 26, 28). Kennedy et al. discovered that elevated serum SP-D levels correlated with more severe lung damage in patients with IPF and SSc-ILD (23), suggesting potential similarities in pulmonary pathophysiology between SSc-ILD and IPF (68). However, it is necessary to acknowledge the inherent heterogeneity among different ILD subtypes in included studies, as well as the limited number of representative studies; these factors may influence our pooled results. Additionally, there is a lack of data concerning ILD occurrence from other ILD subtypes such as drug-induced ILD, primary Sjögren’s syndrome-associated ILD, and sarcoidosis within the pooled studies concerning ILD occurrence. Although data are limited, this meta-analysis encompasses most common ILD as representatives, thereby not significantly affecting the generalizability and reliability.

In addition, pooled results indicated no significant difference in serum SP-D levels between the stable and progressive ILD groups. Considering that the criteria for defining ILD progression have not been standardized in previous studies, a limited number of studies available for pooled analysis during the screening stage may affect the results. The pooling of binary regression analysis demonstrated an association between serum SP-D levels and ILD progression. Zhu et al. highlighted that serum SP-D showed good predictive ability for IPF progression (69), and Györfi et al. identified serum SP-D as a biomarker to detect SSc-ILD progression (70), which were consistent with our results. However, due to the scarcity of studies on SP-D levels and disease progression across other ILD subtypes, further confirmation through more prospective studies utilizing the same definition of ILD progression is necessary.

As a sensitive biomarker reflecting the degree of lung injury, the SP-D level has been used to evaluate AE in chronic lung diseases. Günaydın et al. pointed out higher serum SP-D levels in patients experiencing AE compared with those in stable conditions among the chronic obstructive pulmonary disease (COPD) population (71). In addition, Lomas et al. demonstrated that elevated serum SP-D levels increased the risk of AECOPD (72). Our pooled results showed that AE-ILD patients exhibited higher serum SP-D levels than stable controls; however, the pooled HR results could not support using serum SP-D as a marker for predicting AE-ILD. This limitation may be attributed to the sample size and statistical methodologies employed by included studies; thus, larger prospective studies are needed for further support. Moreover, Kondoha et al. noted no statistically significant differences in serum SP-D levels between AE and non-AE groups in IPF (42). This may stem from retrospective study designs which cannot adequately control the sampling time or detection methods for measuring serum SP-D. Additionally, elevated serum SP-D is recognized as an indicative marker of type II alveolar epithelial injury. A study by Greene et al. found that SP-D levels were significantly elevated during lung injury, peaking on the 7th day (73). Consequently, for patients with AE-ILD, the timing of serum SP-D sampling may also directly influence the outcomes of final statistical analyses. The study by Takeshita presented that combining serum SP-D with thrombin–antithrombin III complex, D-dimer, and plasmin-alpha2 plasmin inhibitor complex can enhance the diagnostic accuracy for AE-ILD (74). These findings speculated that a combined approach utilizing serum SP-D with other biomarkers could provide valuable insights into improving the diagnostic value for AE-ILD.

Finally, our results revealed that serum SP-D levels were significantly higher in the death group compared with the survival group and could predict mortality risk for ILD. Previous studies have identified SP-D as a risk factor for predicting mortality in patients with cardiovascular disease, community-acquired pneumonia, and COPD (75–77). Our findings supported the importance of SP-D in prognostic assessment. Through subgroup analysis and meta-regression, we found that different disease subtypes were significant sources of heterogeneity in the pooled analysis addressing ILD mortality. Although this is consistent with the inherent characteristics of ILD itself, future studies should consider conducting pooled analyses on more refined ILD subtypes and their relationship with SP-D. Additionally, within the IPF subgroup (pooled HR), we observed some intragroup heterogeneity; however, meta-regression analysis showed no significant association between sources of heterogeneity and factors such as age, sample size, male gender, or lung function. We proposed that it might also be related to factors like the timing of SP-D blood sampling, severity of IPF, study design, and other multifactorial aspects in included studies. Hannah et al. mentioned in their pooled analysis that SP-D was not a predictor for IIM-ILD mortality. It is important to highlight that their pooled data only included OR for analysis without exploring HR or WMD regarding IIM-ILD-related mortality (66), which may limit the validity of their conclusions.

SP-D is a key member involved in the regulation of pulmonary inflammation and fibrosis (9, 78), and its serum level can reflect ILD status. While the evaluative value of SP-D across various ILD conditions has been widely recognized, the predictive power of a single marker remains limited. Therefore, combining SP-D with other biomarkers can improve the diagnostic efficiency of ILD status (32, 79), thereby enhancing the predictive capability of SP-D in ILD. Nevertheless, several issues concerning the clinical application of SP-D still need to be addressed, including assessments of antifibrotic efficacy, identification of pulmonary infections, and establishment of reliable cutoff values among different ILD states.

Our study has certain limitations: (1) Due to the strict inclusion and exclusion criteria, as well as challenges in extracting effect sizes from some studies, our pooled analyses were based on a limited number of studies with retrospective design, which may affect our result. It is recommended that high-quality prospective studies be prioritized in the future. Furthermore, the literature should undergo rigorous quality assessment to ensure more reliable results in pooled analyses. Assessment by sensitivity analyses, Egger’s test, and the trim-and-fill method were employed to ensure our results reliable. (2) Given that ILD encompasses a diverse range of pulmonary disorders, our pooled analyses inevitably exhibit varying degrees of heterogeneity. Although our results indicate that SP-D is a potential biomarker for assessing disease status in patients with ILD, the restricted number of included studies hinders us from delineating the role of SP-D in different types of ILD through subgroup analyses, such as CTD-ILD, granulomatous pneumonia, sarcoidosis, and hypersensitivity pneumonitis. As a result, we were unable to thoroughly investigate the differences in SP-D levels among various types of ILD. Future studies should focus on further elucidating the cutoff value for SP-D when predicting different disease states—including occurrence, progression, AE, and mortality—in ILD and its subtypes. This will enhance our understanding concerning the role of SP-D across diverse pathological conditions. (3) The sampling times and detection methods for serum SP-D were not comprehensively detailed in some included studies; this may introduce bias into pooled analyses due to variations in measurement techniques and sampling times. In the future, more research should emphasize the standardization of SP-D sampling times and detection methods. This includes clearly defining specific procedures and methods for SP-D level (e.g., enzyme-linked immunosorbent assay) and establishing reference ranges for normal values. Additionally, it is essential to clarify the optimal timing for serum SP-D sampling—whether before or after drug intervention, during a stable phase, or in the context of an acute exacerbation—to minimize potential impacts on objective results. (4) Two studies included in this study were of moderate quality, which may cause some publication bias. It is worth noting that if the publication bias is substantial, it becomes essential to consider the exclusion of low-quality literature and the screening of studies with negative results in order to mitigate this bias. The results obtained by sensitivity analysis, Egger’s test, and trim-and-fill method support the reliability of our findings.

## Conclusion

Serum SP-D emerges as a promising candidate marker for evaluating the occurrence, progression, AE, and mortality associated with ILD. While our understanding of the relationship between SP-D and ILD has improved significantly, future research based on prospective designs and standardized outcome measures is warranted to elucidate the predictive value of serum SP-D levels concerning disease status in patients with various ILD subtypes. Concurrently, future research should prioritize the standardization of detection methods and sampling times for SP-D, as well as propose the establishment of reference ranges for normal values. Future investigations could explore joint analyses involving SP-D with other potential biomarkers such as KL-6, CA-125, and CA19-9, members of the matrix metalloproteinase family, and chemokine family members, to further enhance the ability of SP-D in assessing disease status among patients with ILD.

## Data Availability

The original contributions presented in the study are included in the article/**Supplementary Material**. Further inquiries can be directed to the corresponding author.
