# Supplementary material for: Serum surfactant protein D as a significant biomarker for predicting occurrence, progression, acute exacerbation, and mortality in interstitial lung disease: a systematic review and meta-analysis
Source: Front Immunol. 2025 Feb 14;16:1450798. doi: 10.3389/fimmu.2025.1450798 (PMC11868069; doi:10.3389/fimmu.2025.1450798)
Supplement: Supplementary file 1 [file DataSheet1.docx]

Table 1. Database and Search strategy

| **Database** | **Search strategy** |
| --- | --- |
| **Pubmed**  **(n=366)** | #1: (((((((((((((Lung Diseases, Interstitial[MeSH Terms]) OR (Diffuse Parenchymal Lung Disease*[Title/Abstract])) OR (Interstitial Lung Disease*[Title/Abstract])) OR (Lung Disease, Interstitial[Title/Abstract])) OR (Pneumonia, Interstitial[Title/Abstract])) OR (Interstitial Pneumonia*[Title/Abstract])) OR (Pneumonias, Interstitial[Title/Abstract])) OR (Pneumonitis, Interstitial[Title/Abstract])) OR (Interstitial Pneumoniti*[Title/Abstract])) OR (Pneumonitides, Interstitial[Title/Abstract]))) )) OR (ILD[Title/Abstract])  = 100150 items |
|  | #2: (((((((Pulmonary Surfactant-Associated Protein D[MeSH Terms]) OR (Pulmonary Surfactant-Associated Protein D[Title/Abstract])) OR (Surfactant Protein D[Title/Abstract])) OR (Pulmonary Surfactant Protein D[Title/Abstract])) OR (Surfactant-Associated Glycoprotein D[Title/Abstract])) OR (Surfactant Associated Glycoprotein D[Title/Abstract])) OR (SP-D[Title/Abstract])) OR (Lung Protein D[Title/Abstract])  = 2406 items |
|  | #3: #1 AND #2 = **366** items |
| **Embase**  **(n=883)** | #1: 'interstitial lung disease'/exp OR 'lung diseases, interstitial':ab,ti OR 'diffuse parenchymal lung disease':ab,ti OR 'interstitial lung disease':ab,ti OR 'interstitial pneumonia':ab,ti OR 'ild':ab,ti  = 151570 items |
|  | #2: 'surfactant protein d'/exp OR 'pulmonary surfactant-associated protein d':ab,ti OR 'surfactant protein d':ab,ti OR 'lung protein d':ab,ti OR 'sp-d':ab,ti  = 4376 items |
|  | #3: #1 AND #2 = **883** items |
| **Cochrane library**  **(n=55)** | #1: (Lung Diseases, Interstitial):ab,ti,kw OR (Diffuse Parenchymal Lung Disease):ab,ti,kw OR (Interstitial Lung Disease):ab,ti,kw OR (Interstitial Pneumonia):ab,ti,kw OR (ILD):ab,ti,kw  = 3575 items |
|  | #2: (Pulmonary Surfactant-Associated Protein D):MeSH OR (Pulmonary Surfactant-Associated Protein D):ab,ti,kw OR (Surfactant Protein D):ab,ti,kw OR (Lung Protein D):ab,ti,kw OR (SP-D):ab,ti,kw  = 1004 items |
|  | #3:#1 AND #2 = **55** items |
| **Web of science**  **(n=365)** | #1: TS= (Lung Diseases, Interstitial OR Diffuse Parenchymal Lung Disease OR Interstitial Lung Disease OR Interstitial Pneumonia OR ILD)  = 25603 items |
|  | #2: TS= (Pulmonary Surfactant-Associated Protein D OR Surfactant Protein D OR Lung Protein D OR SP-D)  = 7194 items |
|  | #3:#1 AND #2 =**365** items |
| **Scopus**  **(n=532)** | #1: TITLE-ABS-KEY ("lung diseases, interstitial" OR "diffuse parenchymal lung disease" OR "interstitial lung disease" OR "interstitial pneumonia" OR "ild")  = 50065 items |
|  | #2: TITLE-ABS-KEY ("pulmonary surfactant-associated protein d" OR "surfactant protein d" OR "lung protein d" OR "sp-d" )  = 4289 items |
|  | #3:#1 AND #2 =**532** items |
| **Ovid**  **(n=198)** | #1:(Lung Diseases, Interstitial or Diffuse Parenchymal Lung Disease or Interstitial Lung Disease or Interstitial Pneumonia or ILD).ti,ab,kw.  = 23432 items |
|  | #2: (Pulmonary Surfactant-Associated Protein D or Surfactant Protein D or Lung Protein D or SP-D).ab,ti,kw.  = 2217 items |
|  | #3:#1 AND #2 =**198** items |

Supplementary Table 2 Results of Newcastle-Ottawa quality assessment Scale for each included study

| **Study** | **Selection** | **Comparability** | **Outcome** | **Total** |
| --- | --- | --- | --- | --- |
| Matama G et al, 2023 | **★★★★** | **★★** | **★★** | 8**★** |
| Lv C et al, 2022 | **★★★★** | **★★** | **★★** | 8**★** |
| Majewski S et al, 2021 | **★★★★** | **★★** | **★★** | 8**★** |
| Kono M et al, 2019 | **★★★★** | **★** | **★★** | 7**★** |
| Papaioannou A I et al, 2016 | **★★★★** | **★★** | **★★** | 8**★** |
| Kennedy B et al, 2015 | **★★★★** | **★** | **★★** | 7**★** |
| Doyle T J et al, 2015 | **★★★★** | **★** | **★★★** | 8**★** |
| Barlo NP et al, 2009 | **★★★★** | **★** | **★★** | 7**★** |
| Yoshikawa S et al, 2007 | **★★★★** | **★** | **★★** | 7**★** |
| Ihn H et al, 2002 | **★★★★** | **★** | **★★** | 7**★** |
| Jee AS et al, 2023 | **★★★★** | **★★** | **★★★** | 9**★** |
| Avouac J et al, 2020 | **★★★★** | **★** | **★★** | 7**★** |
| Sakamoto S et al, 2023 | **★★★★** | **★★** | **★★** | 8**★** |
| Rai M et al, 2023 | **★★★★** | **★★** | **★★★** | 9**★** |
| Yoshikawa T et al, 2020 | **★★★★** | **★★** | **★★** | 8**★** |
| Yamakawa H et al, 2019 | **★★★★** | **★★** | **★★** | 8**★** |
| Ikeda K et al, 2023 | **★★★★** | **★** | **★★** | 7**★** |
| Koda K et al, 2022 | **★★★★** | **★★** | **★★★** | 9**★** |
| Kata Y et al, 2022 | **★★★★** | **★★** | **★★★** | 9**★** |
| Murohashi K et al, 2018 | **★★★★** | **★★** | **★★** | 8**★** |
| Isobe K et al, 2018 | **★★★★** |  | **★★** | 6**★** |
| Kakugawa T et al, 2016 | **★★★★** | **★★** | **★★** | 8**★** |
| Kondoh Y et al, 2015 | **★★★★** | **★★** | **★★★** | 9**★** |
| Collard H R et al, 2010 | **★★★★** | **★★** | **★★** | 8**★** |
| Arai T et al, 2022 | **★★★★** | **★★** | **★★** | 8**★** |
| Arai T et al, 2021 | **★★★★** | **★** | **★★** | 7**★** |
| Domvri K et al, 2023 | **★★★★** | **★** | **★★★** | 8**★** |
| Murohashi K et al, 2019 | **★★★★** | **★** | **★★** | 7**★** |
| Okabayashi H et al, 2017 | **★★★★** | **★★** | **★★** | 8**★** |
| Ikeda S et al, 2015 | **★★★★** | **★★** | **★★** | 8**★** |
| Usui Y et al, 2013 | **★★★★** | **★★** | **★★** | 8**★** |
| Song J W et al, 2013 | **★★★★** | **★★** | **★★** | 8**★** |
| Suzuki Y et al, 2023 | **★★★★** |  | **★★** | 6**★** |
| Arai T et al, 2020 | **★★★★** | **★★** | **★★** | 8**★** |
| Fujisawa T et al, 2019 | **★★★★** | **★** | **★★★** | 8**★** |
| Arai T et al, 2017 | **★★★★** | **★** | **★★** | 7**★** |
| Sokai A et al, 2017 | **★★★★** | **★** | **★★** | 7**★** |
| Nobashi T et al, 2016 | **★★★★** | **★** | **★★** | 7**★** |
| Kohashi Y et al, 2016 | **★★★★** | **★** | **★★** | 7**★** |
| Hamai K et al, 2016 | **★★★★** | **★** | **★★** | 7**★** |
| Hozumi H et al, 2016 | **★★★★** | **★** | **★★** | 7**★** |

Supplementary Table 3 Characteristics of each included study about correlation between SP-D(WMD) and occurrence of ILD

| **No** | **Author, Year** | **Country** | **Study type** | **ILD Type** | **Comparative**  **Group** | **Age(year)** | **nMale** | **nSmoker** | **KL-6**  **(U/ml)** | **FVC%** | **DLCO%** | **Effect Size**  **(n)** | Effect Size  **(ng/ml)** | Detection method of SP-D |
| --- | --- | --- | --- | --- | --- | --- | --- | --- | --- | --- | --- | --- | --- | --- |
| 1 | Matama G et al, 2023 [27] | Japan | retrospective | RA | RA | 66(60-73) | 4(20) | NA | 214.5(177.3-273.0) | NA | NA | 20 | 43.3±37.3^#^ | NA |
|  |  |  |  |  | RA-ILD | 70(65-76) | 6(32) | 6(32) | 1022.0(579.0-1467.0) | 84.4(66.7-107.3) | 61.7±13.9 | 19 | 120.6±122.4^#^ |  |
| 2 | Lv C et al, 2022 [25] | China | retrospective | CTD | CTD | 59.3±12.9 | 8(25.8) | 7(22.6) | NA | NA | NA | 31 | 75.2±17.2 | ELISA |
|  |  |  |  |  | CTD-ILD | 61.9±10.4 | 9(27.3) | 8(24.2) | NA | 84.3±14.3 | 71.4(60.7-86.1) | 33 | 90.4±22.2 |  |
| 3 | Majewski S et al, 2021 [26] | Poland | prospective | IPF | HC | 68.4±6.1 | 10(50) | 11(55) | 464.1(221.4-635.9) | 111.3±20.7 | NA | 20 | 92.2±67.5^#^ | ELISA |
|  |  |  |  |  | IPF | 69.1±7.9 | 17(60.7) | 20(71.4) | 1277.0(727.8-1755) | 75.0±19.3 | 53.3(12.8) | 28 | 355.3±235^#^ |  |
| 4 | Kono M et al, 2019 [24] | Japan | retrospective | IPPFE | IPPFE | 60.2±9.9 | 11(57.9) | 7(36.8) | 405±168 | 71.1±24.1 | 100.0±24.7 | 19 | 173±86 | NA |
|  |  |  |  |  | IPPFE-lower-lobe ILD | 74.3±9.3 | 15(71.4) | 9(42.9) | 626±337 | 59.5±18.6 | 97.6±27.3 | 21 | 217±104 |  |
| 5 | Papaioannou A I et al, 2016 [28] | Greece | cross-sectional | IPF | HC | 64.0(55.5-67.5) | 11(44) | 14(56) | NA | 90.0(82.0-93.5) | 85.0(82.0-88.0) | 25 | 23±28.7^#^ | ELISA |
|  |  |  |  |  | IPF | 72.0(66.8-78.0) | 43(69.4) | 35(56.5) | NA | 68.3(57.9/82.1) | 46.8(35.2-62.9) | 62 | 263.4±103.6^#^ |  |
| 6 | Kennedy B et al, 2015 [23] | Ireland | cross-sectional | SSc | SSc | 52.4±7.9 | 2(40) | 2(40) | 192(0-525) | NA | NA | 5 | 176±82.5^#^ | ELISA |
|  |  |  |  |  | SSc-ILD | 69.7±8.4 | 0(0) | 1(17) | 836(431-1303 ng/ml) | 89.9±18.5 | 37.1±18.2 | 6 | 444.3±512^#^ |  |
| 7 | Doyle T J et al, 2015  (BRASS) [21] | America | prospective | RA | RA | 53±12 | 1(3.4) | 12 (41) | NA | 90±16 | 74±18 | 29 | 11.9±7.9 | ELISA |
|  |  |  |  |  | RA-ILD | 65±10 | 4(23.5) | 9(53) | NA | 70±27 | 57±23 | 17 | 27.5±28.7 |  |
| 7 | Doyle TJ et al, 2015  (ACR) [21] | America | prospective | RA | RA | 50±8 | 6(27.3) | 8 (42) | NA | 101±14 | 84±19 | 22 | 7.1±3.1 | ELISA |
|  |  |  |  |  | RA-ILD | 64±14 | 9(42.9) | 11 (52) | NA | 71±23 | 53±17 | 21 | 31.2±24.1 |  |
| 8 | Barlo NP et al, 2009 [20] | Netherlands | retrospective | IPF | HC | 40.4±11.7 | 115(37.7) | 113(37) | NA | NA | NA | 305 | 58.8±27^#^ | ELISA |
|  |  |  |  |  | IPF | 62.9±12.9 | 56(77.8) | 53(73.6) | NA | 75(60-87) | 43(33-56) | 72 | 373.1±227.7^#^ |  |
| 9 | Yoshikawa S et al, 2007 [29] | Japan | prospective | HP | HC | NA | NA | NA | 265.8±136.2 | NA | NA | 44 | 51.8±29.7 | EIA |
|  |  |  |  |  | HP | NA | NA | NA | 2790.4±2607.6 | NA | NA | 5 | 239.6±184.4 |  |
| 10 | Ihn H et al, 2002 [22] | Japan | cross-sectional | PM/DM | PM/DM | NA | NA | NA | NA | NA | NA | 40 | 38.7±21 | ELISA |
|  |  |  |  |  | PM/DM-ILD | NA | NA | NA | NA | NA | NA | 11 | 118.7±220.2 |  |

^#^: Estimating the sample mean±SD; NA: not applicable; NOS: Newcastle-Ottawa Scale; KL-6 Klebs von den Lungen-6; FVC: forced vital capacity; DLCO: diffusing lung capacity for carbon monoxide; SP-D: surfactant protein-D; EIA: Enzyme Immunoassay; ELISA: enzyme-linked immunosorbent assay; ILD: interstitial lung disease; RA: rheumatoid arthritis; CTD: connective tissue disease; IPF: idiopathic pulmonary fibrosis; HC: healthy control; IPPFE: idiopathic pleuroparenchymal fibroelastosis; SSc: systemic sclerosis; HP: Hypersensitivity Pneumonitis; PM/DM: polymyositis/dermatomyositis

Supplementary Table 4 Characteristics of each included study about correlation between SP-D(OR, multivariable) and occurrence of ILD

| **No** | **Author, Year** | **Country** | **Study type** | **ILD Type** | **Comparative**  **Group** | **Age(year)** | **nMale** | **nSmoker** | **KL-6**  **(U/ml)** | **FVC%** | **DLCO%** | **Sample Size**  **(n)** | Effect Size  **OR (95%CI)** | Detection method of SP-D |
| --- | --- | --- | --- | --- | --- | --- | --- | --- | --- | --- | --- | --- | --- | --- |
| 1 | Jee AS et al, 2023 [31] | Australia | prospective | SSc、IPF | SSc、HC | NA | NA | NA | NA | NA | NA | 209 | 4.21(1.99-8.22) | ML |
|  |  |  |  |  | SSc-ILD、IPF | NA | NA | NA | NA | NA | NA | 431 |  |  |
| 2 | Avouac J et al, 2020 [30] | France/Japan/Switzerland | prospective | RA | RA | 62±12 | 27(25.2) | 28(26) | 376±26 | NA | NA | 107 | 6.16(1.79-21.24) | ELISA |
|  |  |  |  |  | RA-ILD | 71±15 | 18(45) | 24(60) | 961±128 | 79 (28-138) | 61(20-114) | 40 |  |  |

NA: not applicable; NOS: Newcastle-Ottawa Scale; KL-6 Klebs von den Lungen-6; FVC: forced vital capacity; DLCO: diffusing lung capacity for carbon monoxide; ML: magnetic luminex; ELISA: enzyme-linked immunosorbent assay; SSc: systemic sclerosis; HC: healthy control; ILD: interstitial lung disease; IPF: idiopathic pulmonary fibrosis

Supplementary Table 5 Characteristics of each included study about correlation between SP-D(WMD) and progression of ILD

| **No** | **Author, Year** | **Country** | **Study type** | **ILD Type** | **Comparative**  **Group** | **Age(year)** | **nMale** | **nSmoker** | **KL-6**  **(U/ml)** | **FVC%** | **DLCO%** | **Effect Size**  **(n)** | Effect Size  **(ng/ml)** | Detection method of SP-D |
| --- | --- | --- | --- | --- | --- | --- | --- | --- | --- | --- | --- | --- | --- | --- |
| 1 | Sakamoto S et al, 2023 [33] | Japan | retrospective | ANCA | Stable | 69.8±8.0 | 8(35) | 11(48) | 843.9±704.6 | 95.0±16.8 | 68.1±17.2 | 23 | 120.2±89.3 | NA |
|  |  |  |  |  | Progression | 74.5±8.4 | 11(73) | 11(73) | 1158.7±1002.3 | 83.1±22.6 | 68.5±16.0 | 15 | 253.1±227.6 |  |
| 2 | Rai M et al, 2023 [32] | India | prospective | ILD | Stable | 61.0(58.3-67.8) | 4(57.1) | NA | 1068.8 (842.2-1824.0) | 74.0(65.5-79.5) | 55.0(42.3-68.0) | 7 | 185.4±56.2^#^ | ELISA |
|  |  |  |  |  | Progression | 56.0(44.7-69.3) | 8(47.1) | NA | 1357.0(822.6-1543.4) | 70.0(62.0-77.3) | 62.0(47.7-66.3) | 17 | 184.3±46.6^#^ |  |
| 3 | Yoshikawa T et al, 2020 [35] | Japan | retrospective | IPF | Stable | 70(66-76) | 24(75) | 23(71.9) | 885(595.3-1618) | 77.4(65.1-94.4) | 52.8(44.4-57.6) | 32 | 259.5±139.7^#^ | ELISA |
|  |  |  |  |  | Progression | 68(64-75) | 14(82.4) | 13(76.5) | 901(732-1336) | 76.6(66.4-86.9) | 51.7(43.6-70.3) | 17 | 254.7±148.7^#^ |  |
| 4 | Yamakawa H et al, 2019 [34] | Japan | retrospective | ILD | Stable | 59.2±11.50 | 26(41.9) | 33(53.2) | 1856±1319.8 | 76.3±18.31 | 68.6±21.63 | 62 | 261±228.4 | NA |
|  |  |  |  |  | Progression | 64.0±7.65 | 9(69.2) | 9(69.2) | 1456±730.6 | 68.6±15.66 | 67.3±23.14 | 13 | 288±198.2 |  |

^#^: Estimating the sample mean±SD; NA: not applicable; NOS: Newcastle-Ottawa Scale; KL-6 Klebs von den Lungen-6; FVC: forced vital capacity; DLCO: diffusing lung capacity for carbon monoxide; ANCA: Antineutrophil cytoplasmic antibody; ILD: interstitial lung disease; ELISA: enzyme-linked immunosorbent assay; IPF: idiopathic pulmonary fibrosis

Supplementary Table 6 Characteristics of each included study about correlation between SP-D(OR, univariable) and progression of ILD

| **No** | **Author, Year** | **Country** | **Study type** | **ILD Type** | **Comparative**  **Group** | **Age(year)** | **nMale** | **nSmoker** | **KL-6**  **(U/ml)** | **FVC%** | **DLCO%** | **Sample Size**  **(n)** | Effect Size  **OR (95%CI)** | Detection method of SP-D |
| --- | --- | --- | --- | --- | --- | --- | --- | --- | --- | --- | --- | --- | --- | --- |
| 1 | Sakamoto S et al, 2023 [33] | Japan | retrospective | ANCA | Stable | 69.8±8.0 | 8(35) | 11(48) | 843.9±704.6 | 95.0±16.8 | 68.1±17.2 | 23 | 1.006(1-1.012) | NA |
|  |  |  |  |  | Progression | 74.5±8.4 | 11(73) | 11(73) | 1158.7±1002.3 | 83.1±22.6 | 68.5±16.0 | 15 |  |  |
| 2 | Ikeda K et al, 2023(Pirfenidone) [36] | Japan | prospective | IPF | Stable | NA | NA | NA | NA | NA | NA | 41 | 1.004(1.002-1.006) | ELISA |
|  |  |  |  |  | Progression | NA | NA | NA | NA | NA | NA | 117 |  |  |
| 2 | Ikeda K et al, 2023(Placebo) [36] | Japan | prospective | IPF | Stable | NA | NA | NA | NA | NA | NA | 38 | 1.001(0.999-1.002) | ELISA |
|  |  |  |  |  | Progression | NA | NA | NA | NA | NA | NA | 65 |  |  |

NA: not applicable; NOS: Newcastle-Ottawa Scale; KL-6 Klebs von den Lungen-6; FVC: forced vital capacity; DLCO: diffusing lung capacity for carbon monoxide; ANCA: Antineutrophil cytoplasmic antibody; ILD: interstitial lung disease; ELISA: enzyme-linked immunosorbent assay; IPF: idiopathic pulmonary fibrosis

Supplementary Table 7 Characteristics of each included study about correlation between SP-D(WMD) and AE of ILD

| **No** | **Author, Year** | **Country** | **Study type** | **ILD Type** | **Comparative**  **Group** | **Age(year)** | **nMale** | **nSmoker** | **KL-6**  **(U/ml)** | **FVC%** | **DLCO%** | **Effect Size**  **(n)** | Effect Size  **(ng/ml)** | Detection method of SP-D |
| --- | --- | --- | --- | --- | --- | --- | --- | --- | --- | --- | --- | --- | --- | --- |
| 1 | Koda K et al, 2022 [41] | Japan | prospective | ILD | Non-AE | 72(66-77) | 75(87.2) | NA | 524.3(383-1091) | 82.8(70.2-97.1) | 73.3(57.8-83.2) | 86 | 102.7±73.1^#^ | NA |
|  |  |  |  |  | AE | 70(65-74) | 23(100) | NA | 644(422-1201) | 72.4(59.8-84.0) | 75.2(62.7-81.8) | 23 | 132.1±97.2^#^ |  |
| 2 | Kata Y et al, 2022 [40] | Japan | prospective | ILD | Non-AE | 69(62-77) | 7(30) | NA | 905(553-1956) | NA | NA | 23 | 179.2±49.8^#^ | NA |
|  |  |  |  |  | AE | 72(66-77) | 3(33) | NA | 1249(657-2358) | NA | NA | 9 | 219.9±120.7^#^ |  |
| 3 | Murohashi K et al, 2018 [43] | Japan | retrospective | ILD | Non-AE | 70(63-76) | 8(57) | NA | 977.0(354.8-2049.3) | NA | NA | 14 | 204.5±135.5^#^ | NA |
|  |  |  |  |  | AE | 76 (66-80) | 9(64) | NA | 1056(720.0-2454.5) | NA | NA | 14 | 225.2±180.4^#^ |  |
| 4 | Isobe K et al, 2018 [38] | Japan | retrospective | ILD | Non-AE | NA | NA | NA | 926.4±862.3 | 90.7±12.6 | 62.3±17.5 | 79 | 121.7±10.1 | NA |
|  |  |  |  |  | AE | NA | NA | NA | 770.1±556.9 | 91.0±13.1 | 61.6±19.4 | 30 | 131.2±21.2 |  |
| 5 | Kakugawa T et al, 2016 [39] | Japan | retrospective | IPF | Non-AE | 69.0(62.0-73.0) | 31(75.6) | 32(78) | 912(595.0-1802.0) | 80.4(59.0-93.9) | 58.0(46.1-72.5) | 41 | 226.8±162.1^#^ | NA |
|  |  |  |  |  | AE | 68.0(62.0-73.5) | 20(83.3) | 20(83.3) | 1258(744.5-2006.0) | 70.5(59.9-82.1) | 46.2(41.0-72.3) | 24 | 263.1±129.6^#^ |  |
| 6 | Kondoh Y et al, 2015 [42] | Japan | retrospective | IPF | Non-AE | 64.9±6.8 | 203(80.2) | 203(80.2) | 1321±805 | 78.2±17.4 | 53.7±18.0 | 253 | 93.3±52.2 | NA |
|  |  |  |  |  | AE | 64.0±9.3 | 10(71.4) | 9(64.3) | 1237±729 | 70.3±16.3 | 52.7±14.1 | 14 | 90.0±40.6 |  |
| 7 | Collard H R et al, 2010 [37] | South Korea | retrospective | IPF | Non-AE | 63(7) | 16(80) | 18(90) | 895(598-1428) | 84(19) | 74(22) | 20 | 250.1±170.7^#^ | ELISA |
|  |  |  |  |  | AE | 66(8) | 36(77) | 40(85) | 1791(1155-2866) | 75(18) | 64(20) | 47 | 393.6±273.8^#^ |  |

^#^: Estimating the sample mean±SD; AE: acute exacerbation; NA: not applicable; NOS: Newcastle-Ottawa Scale; KL-6 Klebs von den Lungen-6; FVC: forced vital capacity; DLCO: diffusing lung capacity for carbon monoxide; ILD: interstitial lung disease; ELISA: enzyme-linked immunosorbent assay; IPF: idiopathic pulmonary fibrosis

Supplementary Table 8 Characteristics of each included study about correlation between SP-D(HR, univariable) and AE of ILD

| **No** | **Author, Year** | **Country** | **Study type** | **ILD Type** | **Comparative**  **Group** | **Age(year)** | **nMale** | **nSmoker** | **KL-6**  **(U/ml)** | **FVC%** | **DLCO%** | **Sample Size**  **(n)** | Effect Size  **HR (95%CI)** | Detection method of SP-D |
| --- | --- | --- | --- | --- | --- | --- | --- | --- | --- | --- | --- | --- | --- | --- |
| 1 | Arai T et al, 2022 [44] | Japan | retrospective | IPF | Non-AE | NA | NA | NA | NA | NA | NA | 48 | 1.017(1.001-1.034) | ELISA |
|  |  |  |  |  | AE | NA | NA | NA | NA | NA | NA | 23 |  |  |
| 2 | Arai T et al, 2021 [45] | Japan | retrospective | IIP | Non-AE | NA | NA | NA | NA | NA | NA | 176 | 1.005(0.998-1.012) | ELISA |
|  |  |  |  |  | AE | NA | NA | NA | NA | NA | NA | 45 |  |  |
| 3 | Kondoh Y et al, 2015 [42] | Japan | retrospective | IPF | Non-AE | 64.9±6.8 | 203(80.2) | 203(80.2) | 1321±805 | 78.2±17.4 | 53.7±18.0 | 253 | 1.000(0.997-1.004) | NA |
|  |  |  |  |  | AE | 64.0±9.3 | 10(71.4) | 9(64.3) | 1237±729 | 70.3±16.3 | 52.7±14.1 | 14 |  |  |

NA: not applicable; NOS: Newcastle-Ottawa Scale; KL-6 Klebs von den Lungen-6; FVC: forced vital capacity; DLCO: diffusing lung capacity for carbon monoxide; ANCA: Antineutrophil cytoplasmic antibody; ILD: interstitial lung disease; ELISA: enzyme-linked immunosorbent assay; IPF: idiopathic pulmonary fibrosis; IIP: idiopathic interstitial pneumonia

Supplementary Table 9 Characteristics of each included study about correlation between SP-D(WMD) and mortality of ILD

| **No** | **Author, Year** | **Country** | **Study type** | **ILD Type** | **Comparative**  **Group** | **Age(year)** | **nMale** | **nSmoker** | **KL-6**  **(U/ml)** | **FVC%** | **DLCO%** | **Effect Size**  **(n)** | Effect Size  **(ng/ml)** | Detection method of SP-D |
| --- | --- | --- | --- | --- | --- | --- | --- | --- | --- | --- | --- | --- | --- | --- |
| 1 | Rai M et al, 2023 [32] | India | prospective | ILD | Survival | 56.0(49.0–69.0) | 18(52.94) | NA | 1083.4(856.2–1668.5) | 70.0 (62.0–78.0) | 60.5 (48.0–66.1) | 34 | 177.9±38.5^#^ | ELISA |
|  |  |  |  |  | Death | 76.0(71.0–84.0) | 5(71.4) | NA | 3990.4(3490.0–4467.6) | 42.0 (42.0–42.0) | 40.0 (37.2–40.0) | 7 | 243.1±38.7^#^ |  |
| 2 | Domvri K et al, 2023 [46] | Greece | prospective | IPF | Survival | 71±5 | 30(75) | 34(85) | 618±184 | 78.7±14 | 50.3±11.7 | 40 | 40±25.5 | ELISA |
|  |  |  |  |  | Death | 74±6 | 30(94) | 30(94) | 684±161 | 69.8±20.6 | 41.1±12 | 32 | 56.3±19.7 |  |
| 3 | Murohashi K et al, 2019 [48] | Japan | retrospective | IIP | Survival | 75.0(71.5–80.8) | 30(63) | NA | 955(574–1835) | NA | NA | 48 | 279.7±185^#^ | NA |
|  |  |  |  |  | Death | 76.5(72.3–81.5) | 19(95) | NA | 897(573–1718) | NA | NA | 20 | 381.8±419.7^#^ |  |
| 4 | Okabayashi H et al, 2017 [49] | Japan | retrospective | DM | Survival | 64(36–75) | 1(20) | 1(20) | 906(390–2725) | NA | NA | 5 | 210.9±224.6^#^ | NA |
|  |  |  |  |  | Death | 58(44–77) | 2(22.2) | 2(22.2) | 2024(403–4730) | NA | NA | 9 | 150.3±285.2^#^ |  |
| 5 | Ikeda S et al, 2015 [47] | Japan | retrospective | DM | Survival | 58.5(47.8–64.3) | 2(50) | NA | 1135 (930–1190) | NA | NA | 4 | 48.9±56.9^#^ | NA |
|  |  |  |  |  | Death | 63.0(59.3–64.5) | 4(66.7) | NA | 524(470–626) | NA | NA | 6 | 32.5±15.9^#^ |  |
| 6 | Usui Y et al, 2013 [51] | Japan | retrospective | ILD | Survival | 69±5 | 14(73.7) | 15(78.9) | 1535±1013 | NA | NA | 19 | 360±262 | NA |
|  |  |  |  |  | Death | 72±10 | 23(71.9) | 23(71.9) | 1513±687 | NA | NA | 32 | 427±321 |  |
| 7 | Song J W et al, 2013 [50] | South Korea | retrospective | IPF | Survival | 60.9±7.4 | 46(85.2) | 39(72.2) | 1099.0±796.1 | 79.2±15.3 | 71.9±18.5 | 54 | 184.8±139.6 | ELISA |
|  |  |  |  |  | Death | 63.5±8.7 | 49(76.6) | 49(76.6) | 1851.7±1268.4 | 71.6±18.2 | 58.9±16.7 | 64 | 253.6±287.5 |  |

^#^: Estimating the sample mean±SD; NA: not applicable; NOS: Newcastle-Ottawa Scale; KL-6 Klebs von den Lungen-6; FVC: forced vital capacity; DLCO: diffusing lung capacity for carbon monoxide; ELISA: enzyme-linked immunosorbent assay; ILD: interstitial lung disease; idiopathic interstitial pneumonia; IPF: idiopathic pulmonary fibrosis; IIP: idiopathic interstitial pneumonia; DM: dermatomyositis

Supplementary Table 10 Characteristics of each included study about correlation between SP-D(HR, univariable) and mortality of ILD

| **No** | **Author, Year** | **Country** | **Study type** | **ILD Type** | **Comparative**  **Group** | **Age(year)** | **nMale** | **nSmoker** | **KL-6**  **(U/ml)** | **FVC%** | **DLCO%** | **Sample Size**  **(n)** | Effect Size  **HR (95%CI)** | Detection method of SP-D | NOS  Score |
| --- | --- | --- | --- | --- | --- | --- | --- | --- | --- | --- | --- | --- | --- | --- | --- |
| 1 | Suzuki Y et al, 2023 [60] | Japan | retrospective | IPPFE | Survival | NA | NA | NA | NA | NA | NA | 54 | 1.001(1.000-1.002) | NA | 6**★** |
|  |  |  |  |  | Death | NA | NA | NA | NA | NA | NA | 77 |  |  |  |
| 2 | Arai T et al, 2022 [44] | Japan | retrospective | IPF | Survival | NA | NA | NA | NA | NA | NA | 39 | 1.020(1.005-1.036) | ELISA | 8**★** |
|  |  |  |  |  | Death | NA | NA | NA | NA | NA | NA | 32 |  |  |  |
| 3 | Arai T et al, 2020 [52] | Japan | retrospective | IIP | Survival | NA | NA | NA | NA | NA | NA | NA | 1.018(1.003-1.032) | ELISA | 8**★** |
|  |  |  |  |  | Death | NA | NA | NA | NA | NA | NA | NA |  |  |  |
| 4 | Fujisawa T et al, 2019 [54] | Japan | retrospective | DM | Survival | 54.5(31–69) | 4(20) | NA | 660(249–2450) | 79.3(36.6–125.5) | NA | 20 | 1(0.988-1.02) | NA | 8**★** |
|  |  |  |  |  | Death | 54(32–80) | 3(30) | NA | 1026(297–2230) | 51.3(38.1–82.8) | NA | 10 |  |  |  |
| 5 | Arai T et al, 2017 [53] | Japan | retrospective | IIP | Survival | NA | NA | NA | NA | NA | NA | 28 | 1.008(1.001-1.014) | ELISA | 7**★** |
|  |  |  |  |  | Death | NA | NA | NA | NA | NA | NA | 56 |  |  |  |
| 6 | Sokai A et al, 2017 [59] | Japan | retrospective | IPF | Survival | NA | NA | NA | NA | NA | NA | 53 | 1.01(0.99-1.02) | EIA | 9**★** |
|  |  |  |  |  | Death | NA | NA | NA | NA | NA | NA | 22 |  |  |  |
| 7 | Nobashi T et al, 2016 [58] | Japan | retrospective | ILD | Survival | NA | NA | NA | NA | NA | NA | NA | 1.00(1.00-1.01) | NA | 7**★** |
|  |  |  |  |  | Death | NA | NA | NA | NA | NA | NA | NA |  |  |  |
| 8 | Kohashi Y et al, 2016 [57] | Japan | retrospective | IPF | Survival | NA | NA | NA | NA | NA | NA | 31 | 1.021(1.003-1.04) | ELISA | 8**★** |
|  |  |  |  |  | Death | NA | NA | NA | NA | NA | NA | 16 |  |  |  |
| 9 | Hamai K et al, 2016 [55] | Japan | retrospective | IPF | Survival | NA | NA | NA | NA | NA | NA | NA | 1.000(0.998-1.002) | ELISA | 8**★** |
|  |  |  |  |  | Death | NA | NA | NA | NA | NA | NA | NA |  |  |  |
| 10 | Hozumi H et al, 2016 [56] | Japan | retrospective | PM/DM | Survival | NA | NA | NA | NA | NA | NA | 47 | 0.999(0.994-1.002) | NA | 8**★** |
|  |  |  |  |  | Death | NA | NA | NA | NA | NA | NA | 13 |  |  |  |
| 11 | Usui Y et al, 2013 [51] | Japan | retrospective | ILD | Survival | 69±5 | 14(73.7) | 15(78.9) | 1535±1013 | NA | NA | 19 | 1.001(0.999-1.002) | NA | 8**★** |
|  |  |  |  |  | Death | 72±10 | 23(71.9) | 23(71.9) | 1513±687 | NA | NA | 32 |  |  |  |
| 12 | Song JW et al, 2013 [50] | South Korea | retrospective | IPF | Survival | 60.9±7.4 | 46(85.2) | 39(72.2) | 1099.0±796.1 | 79.2±15.3 | 71.9±18.5 | 54 | 1.002(1.001-1.003) | ELISA | 8**★** |
|  |  |  |  |  | Death | 63.5±8.7 | 49(76.6) | 49(76.6) | 1851.7±1268.4 | 71.6±18.2 | 58.9±16.7 | 64 |  |  |  |

NA: not applicable; NOS: Newcastle-Ottawa Scale; KL-6 Klebs von den Lungen-6; FVC: forced vital capacity; DLCO: diffusing lung capacity for carbon monoxide; ILD: interstitial lung disease; EIA: Enzyme Immunoassay; ELISA: enzyme-linked immunosorbent assay; IPF: idiopathic pulmonary fibrosis; IIP: idiopathic interstitial pneumonia; PM/DM: polymyositis/dermatomyositis

Supplementary Table 11 Meta-regression analysis for the assessment of heterogeneity sources in pooled studies associated with IPF mortality (HR)

|  | Age | BMI | Country | Sample size | ILD type | PR time | Male% | FVC% | DLCO% |
| --- | --- | --- | --- | --- | --- | --- | --- | --- | --- |
| Tao^2^ | 0.381 | 0.171 | 0.274 | 0.508 | 0.559 | 0.505 | 0.325 | 0.083 | 0.041 |
| I^2^% | 29.68 | 16.80 | 35.40 | 50.25 | 55.04 | 52.71 | 31.47 | 21.92 | 1.07 |
| Adj R^2^% | -17.93 | 24.86 | 45.31 | -1.38 | -11.63 | -0.70 | 19.69 | 69.14 | -8.95 |
| t | 0.94 | 0.62 | -1.93 | -0.59 | -0.31 | -0.87 | -0.63 | 1.63 | -0.35 |
| *P* value | 0.364 | 0.547 | 0.072 | 0.565 | 0.764 | 0.395 | 0.548 | 0.126 | 0.73 |

BMI: body mass index; ILD: interstitial lung disease; PR: pulmonary rehabilitation; FVC: forced vital capacity; DLCO: diffusing lung capacity for carbon monoxide.

Supplementary Figure 1 Plot for the assessment of heterogeneity among the included studies((non-ILD and HC) vs ILD) through One-by-one elimination method


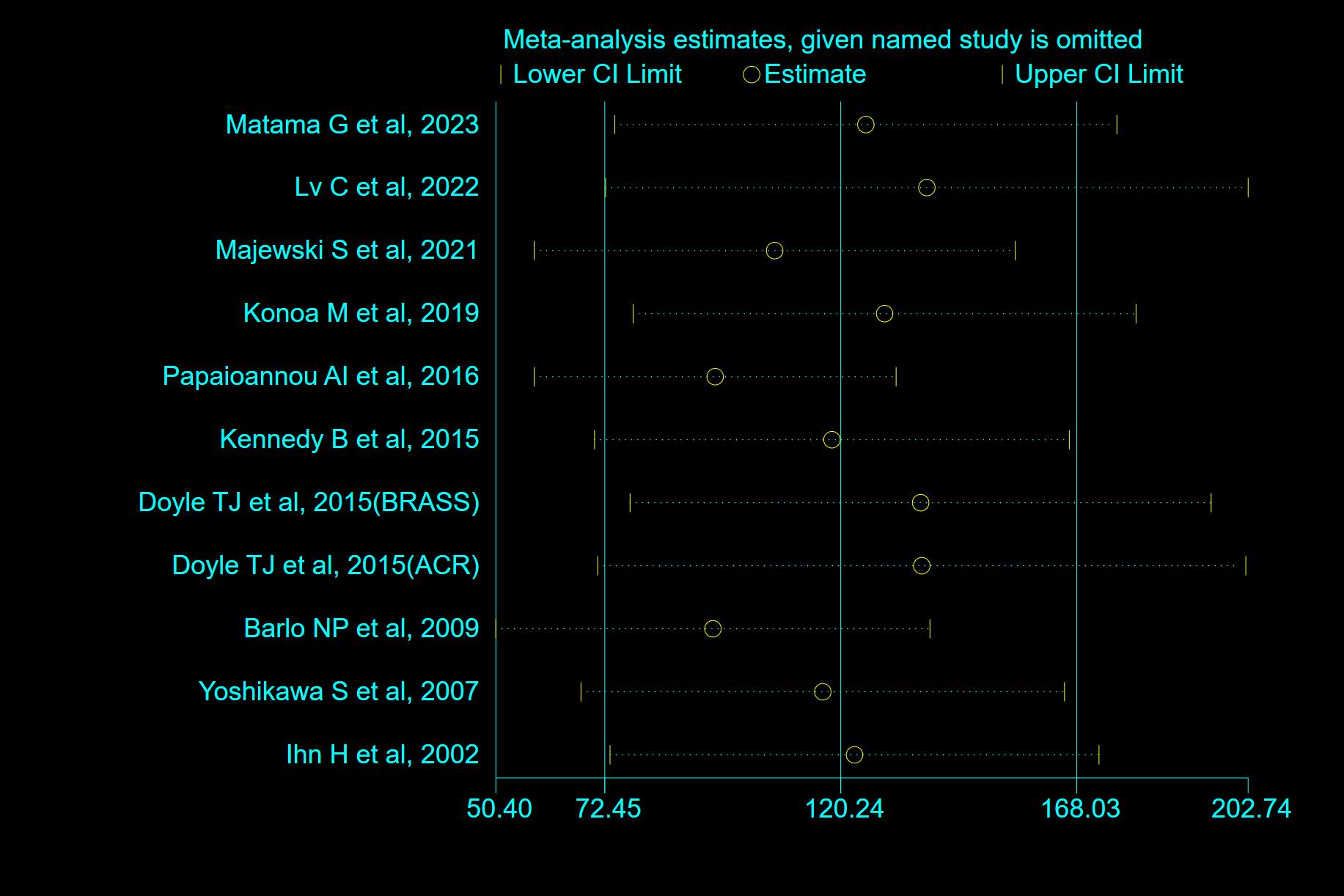


Supplementary Figure 2 Meta-regression analysis for the assessment of heterogeneity sources in pooled studies associated with ILD occurrence


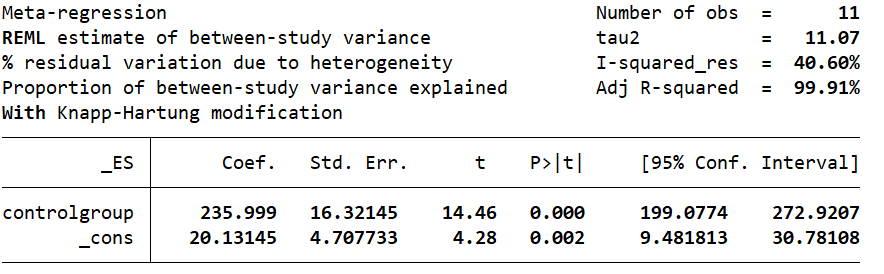


The control groups were categorized into two types: 1) healthy populations versus patients with ILD; 2) disease control group without ILD versus those with ILD (including rheumatoid arthritis, connective tissue disease, idiopathic pleuroparenchymal fibroelastosis, systemic sclerosis and polymyositis/dermatomyositis). ILD: interstitial lung disease.

Supplementary Figure 3 Plot for the assessment of heterogeneity among the included studies(ILD Occurrence(OR, multivariable)) through One-by-one elimination method


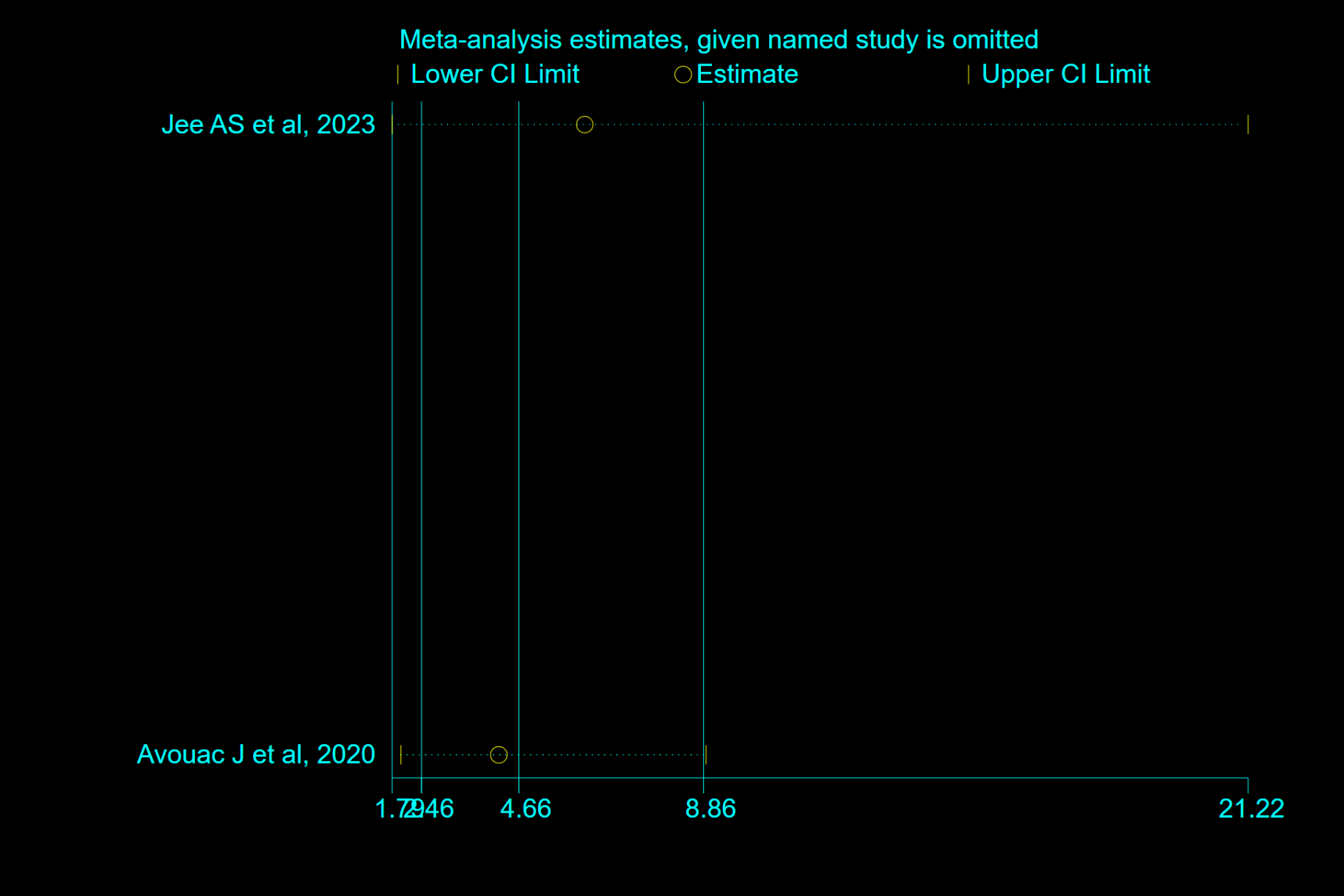


ILD: interstitial lung disease; OR: odds ratio.

Supplementary Figure 4 Plot for the assessment of heterogeneity among the included studies(Stable vs Progression(WMD)) through One-by-one elimination method


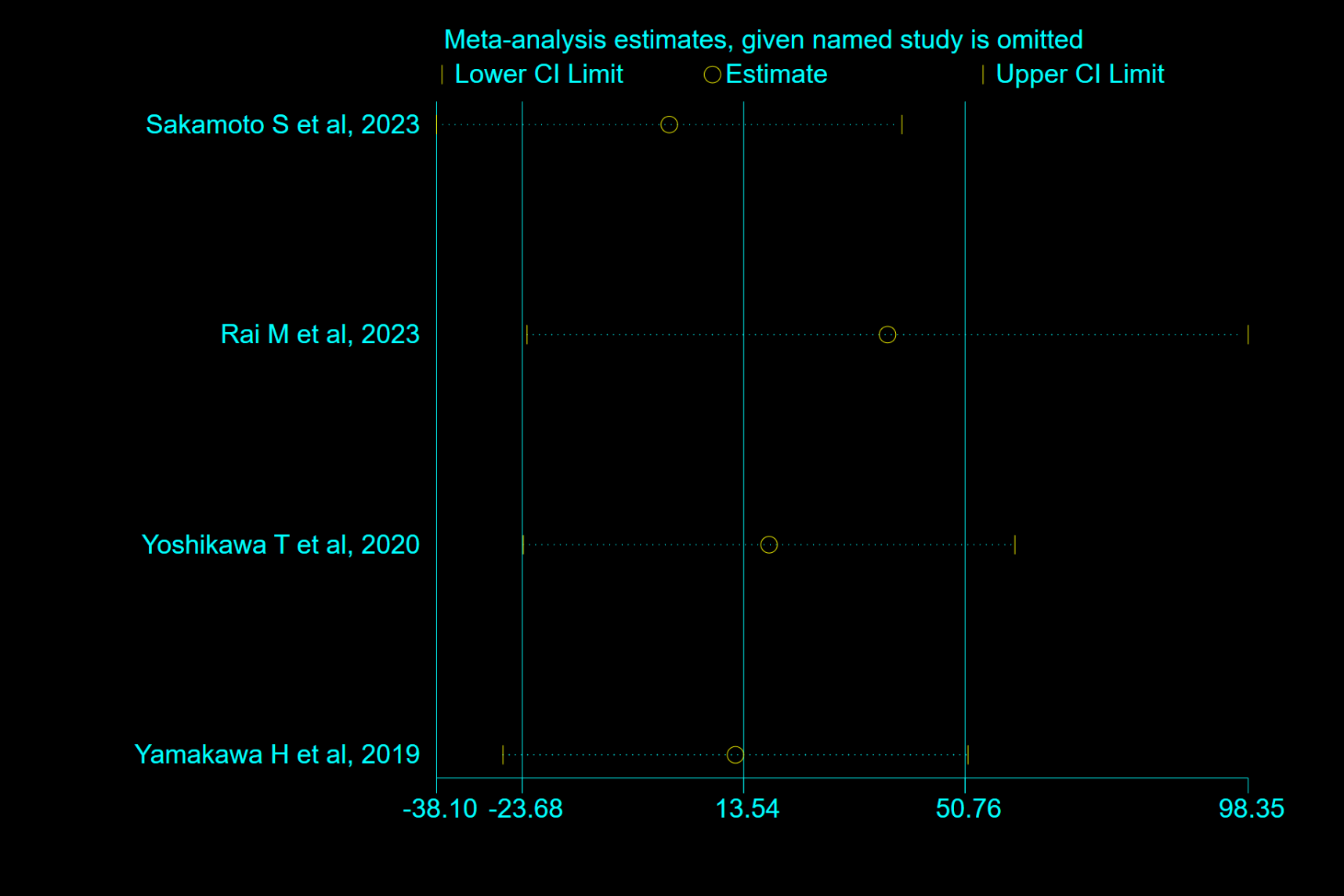


WMD: weighted mean difference.

Supplementary Figure 5 Plot for the assessment of heterogeneity among the included studies(Progression (OR, univariable)) through One-by-one elimination method


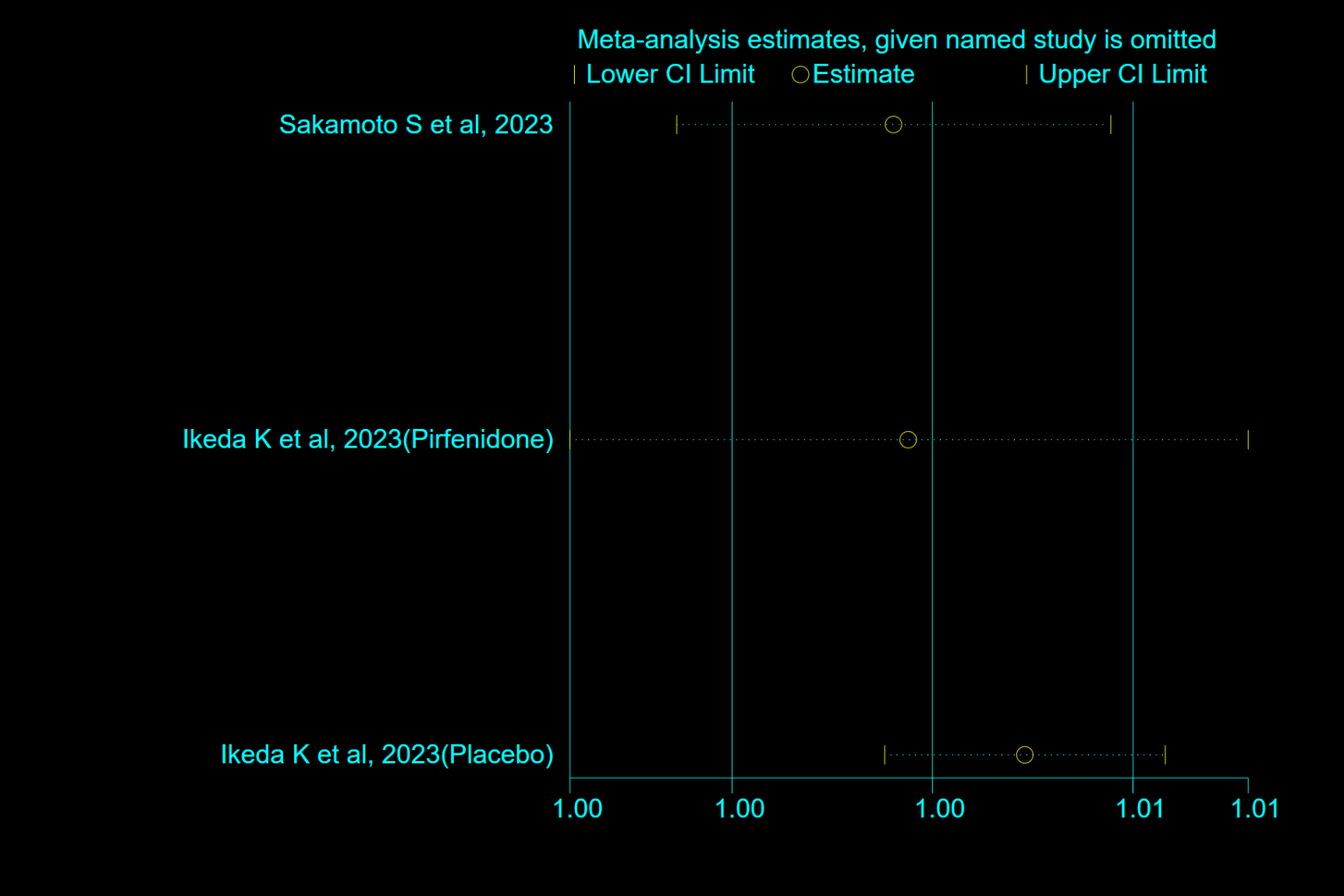


OR: odds ratio.

Supplementary Figure 6 Plot for the assessment of heterogeneity among the included studies(Non-AE vs AE(WMD)) through One-by-one elimination method


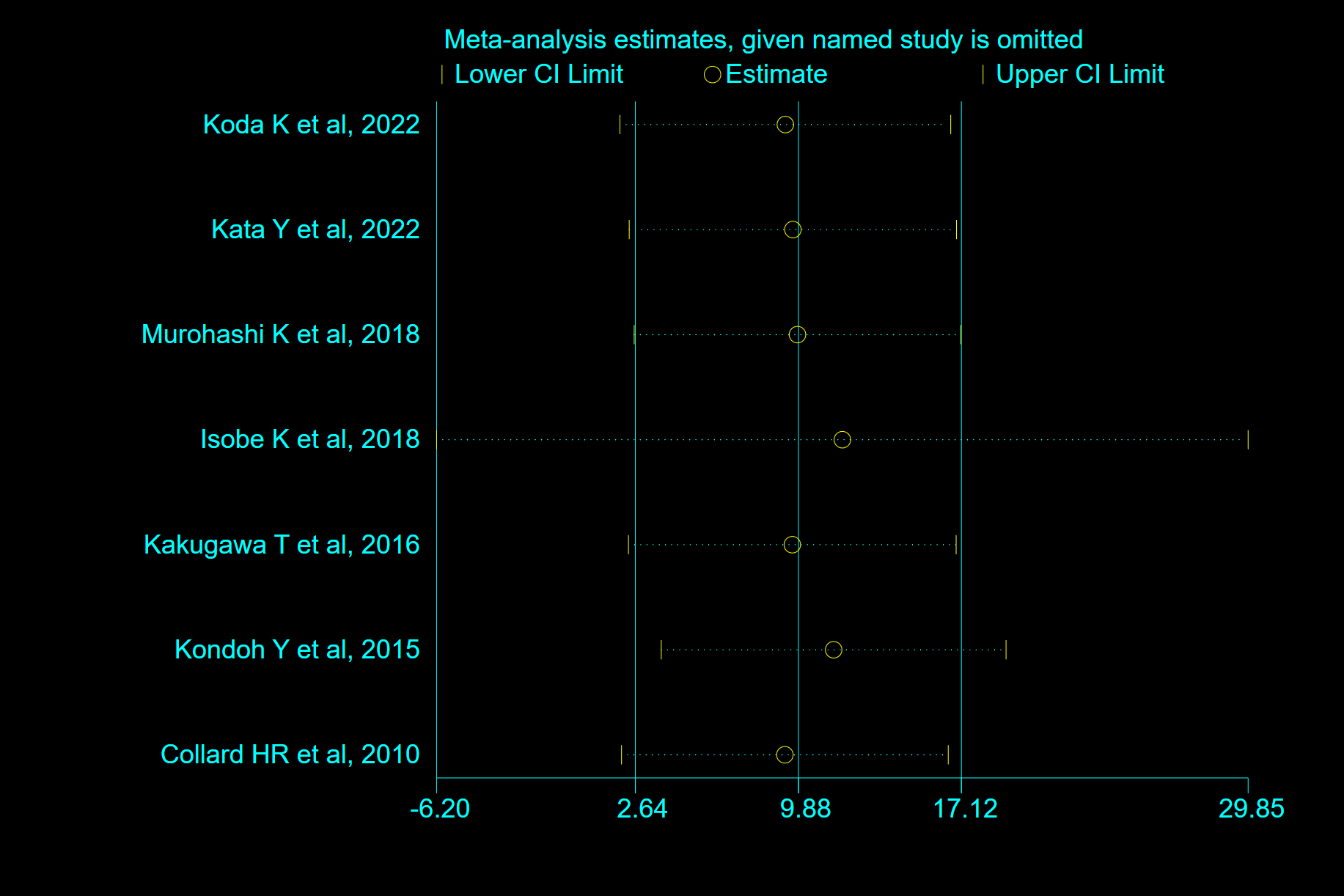


WMD: weighted mean difference; AE: acute exacerbation.

Supplementary Figure 7 Plot for the assessment of heterogeneity among the included studies(AE(HR, univariable)) through One-by-one elimination method


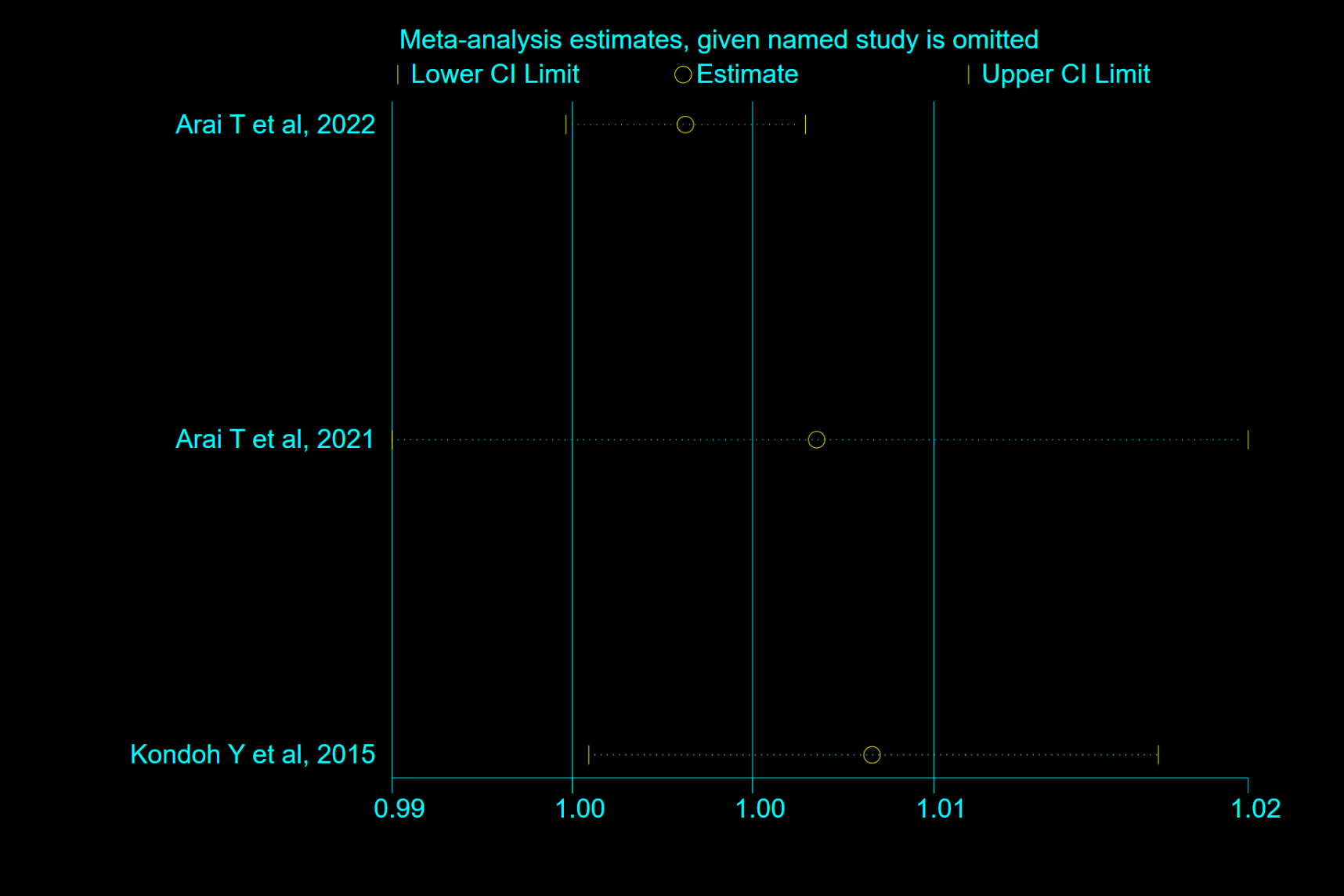


HR: hazard ratio; AE: acute exacerbation.

Supplementary Figure 8 Plot for the assessment of heterogeneity among the included studies(Survival vs Death(WMD)) through One-by-one elimination method


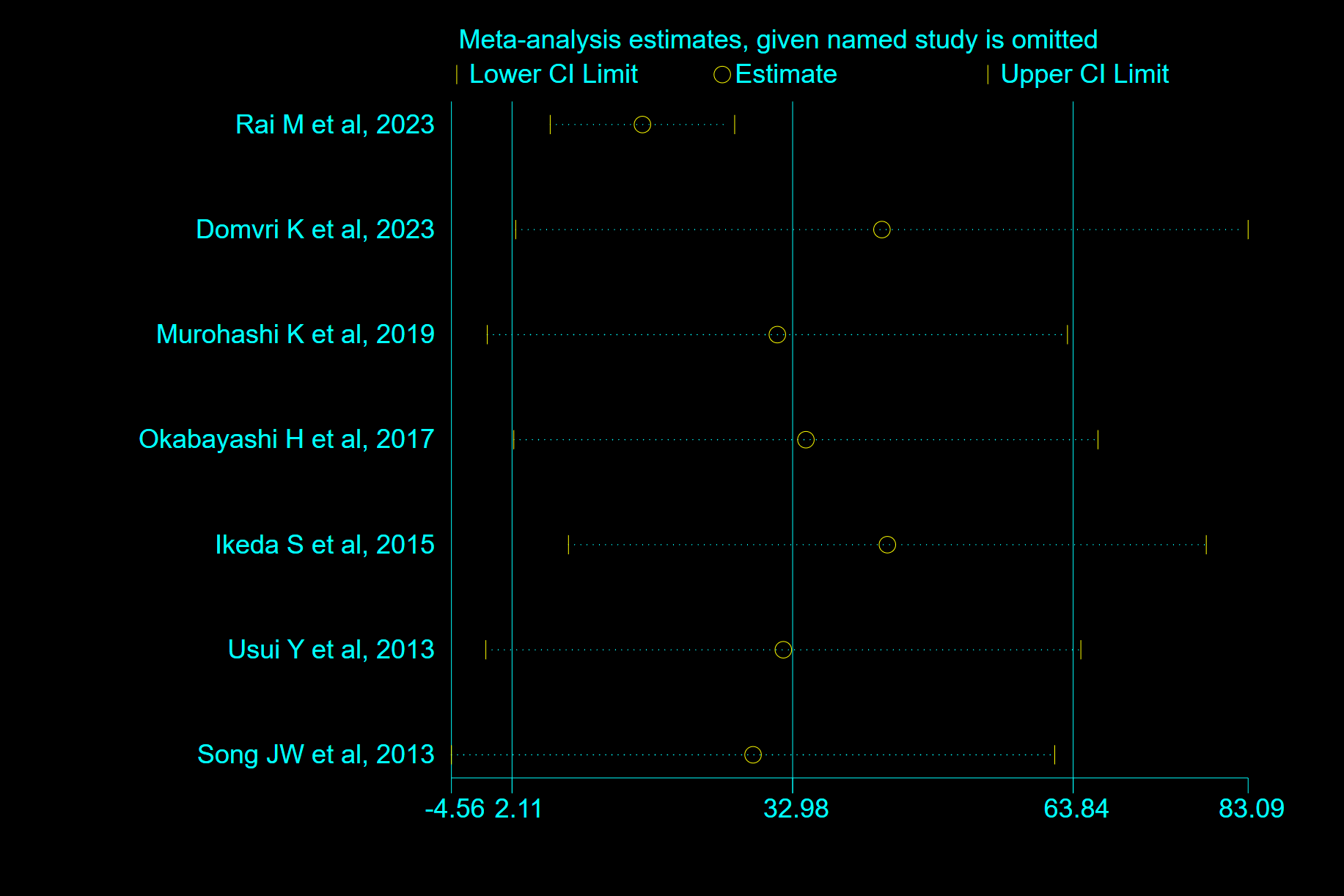


WMD: weighted mean difference.

Supplementary Figure 9 Meta-regression analysis for the assessment of heterogeneity sources in pooled studies associated with ILD mortality (WMD)


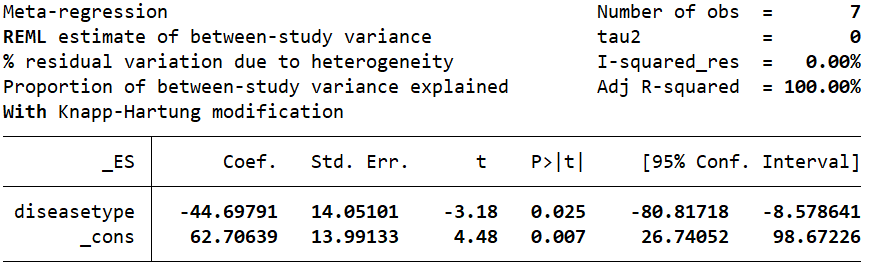


According to the ILD subtypes, the ILD group was divided into three subgroups: ILD group, IIP group including IPF, and DM-ILD group.

ILD: interstitial lung disease; IIP: idiopathic interstitial pneumonitis; IPF: idiopathic pulmonary fibrosis; DM-ILD: dermatomyositis-associated interstitial lung disease; WMD: weighted mean difference.

Supplementary Figure 10 Plot for the assessment of heterogeneity among the included studies(Mortality(HR, univariable)) through One-by-one elimination method


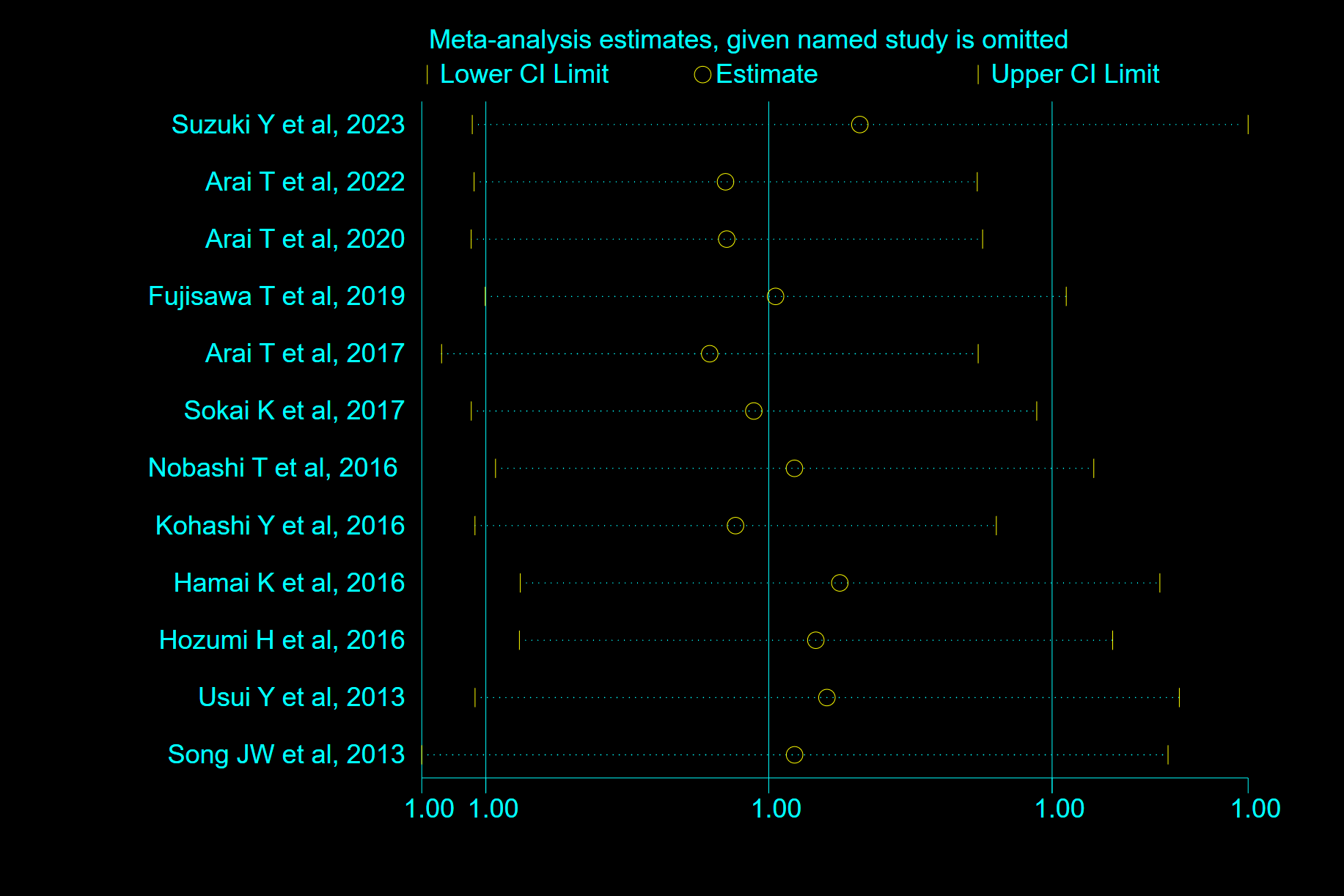


HR: hazard ratio.
